# Adsorption‐Engineered Hydrocarbon Ionomers for Durable Proton‐Exchange Membrane Fuel Cells

**DOI:** 10.1002/adma.74561

**Published:** 2026-08-12

**Authors:** Heemin Park, Kate Chen, Su Min Ahn, Hengquan Guo, Cy Fujimoto, Jong‐Ho Choi, Lynda Amichi, Danah Kim, Seung Geol Lee, Xiaojing Wang, Jacob S. Spendelow, Sun Young Kang, Panagiotis Bexis, Eun Joo S. Park, Yu Seung Kim

**Affiliations:** ^1^ MPA‐11 Los Alamos National Laboratory Los Alamos New Mexico USA; ^2^ Department of Materials Science and Engineering Ulsan National Institute of Science and Technology (UNIST) Ulsan Republic of Korea; ^3^ Nanoscale Sciences Department Sandia National Laboratories Albuquerque New Mexico USA; ^4^ Department of Electrical and Energy Engineering Kyungil Univeristy Gyeongbuk Republic of Korea; ^5^ Center for Nanophase Materials Sciences Oak Ridge National Laboratory Oak Ridge Tennessee USA; ^6^ Graduate School of Semiconductor Materials and Devices Engineering Ulsan National Institute of Science and Technology (UNIST) Ulsan Republic of Korea

**Keywords:** durability, electrode, ionomer, ionomer adsorption, materials science, proton exchange membrane fuel cell, sulfonic acid

## Abstract

Reducing reliance on perfluoroalkyl substances (PFAS) in proton‐exchange membrane fuel cells requires hydrocarbon ionomers that combine high performance with long‐term durability, a persistent challenge in catalyst‐layer design. Here, we identify oxidation‐driven ionomer‐catalyst interfacial degradation as a dominant failure pathway in hydrocarbon ionomer‐bonded cathodes and introduce an adsorption‐engineering strategy to overcome this limitation. The comparison of a commercial sulfonated poly(phenylene) (Pemion) with structurally engineered sulfonated poly(fluorene)s demonstrated that electrode durability is governed by the interplay between ionomer adsorption strength and resistance to oxidative degradation on carbon‐supported Pt catalysts. A poly(fluorene) ionomer with mobile alkyl sulfonic acid groups forms resilient interfaces, delivering 1.28 A cm^−^^2^ at 0.65 V under fully humidified H_2_/air conditions (80°C and 150 kPa_abs_), comparable to Pemion. After 90,000 accelerated potential cycles, the poly(fluorene)‐bonded cathode exhibits significantly improved durability, with only 29% performance loss compared to 58% for Pemion; further molecular refinement reduces the loss to 17%, approaching that of Nafion‐bonded cathodes (14%). These findings establish adsorption‐engineered ionomer design that decouples interfacial anchoring from oxidative degradation as a general strategy for achieving durable, high‐performance PFAS‐free PEM fuel cell electrodes.

## Graphical abstract

Hydrocarbon ionomers suffer from oxidation‐driven interfacial degradation in PEM fuel cell cathodes, leading to catalyst instability. Here, adsorption‐engineered poly(fluorene) ionomers decouple interfacial anchoring from oxidative degradation, enabling strong catalyst–ionomer interactions while resisting electrochemical oxidation. This strategy delivers high performance and significantly enhanced durability, providing a general design principle for PFAS‐free PEM fuel cell electrodes.

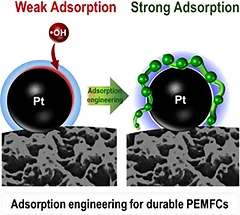

## 1 Introduction

The development of hydrocarbon‐based ionomers that combine high electrochemical performance with long‐term durability remains a central challenge in fuel cell technology. As both membrane separators and ionomeric binders in catalyst layers, ionomers play a critical role in determining the efficiency, stability, and scalability of proton‐exchange membrane fuel cells (PEMFCs) [1, 2]. This challenge has gained renewed urgency in light of increasing environmental and regulatory pressures to reduce reliance on perfluoroalkyl substances (PFAS), such as Nafion, which continue to dominate PEMFC applications despite their environmental persistence and ecological impact [3].

Over the past several decades, extensive efforts have been devoted to replacing PFAS‐based ionomeric binders with hydrocarbon alternatives. Only recently, however, have hydrocarbon ionomers begun to achieve performance metrics comparable to their perfluorosulfonic acid (PFSA) counterparts. Poly(norbornene)‐based ionomers have enabled peak power densities up to 3.5 W cm^−2^ in anion‐exchange membrane fuel cells (AEMFCs) [4], while poly(fluorene)‐ and poly(phenylene)‐based ionomers have demonstrated promising performance in both AEMFCs and PEMFCs [5, 6, 7, 8]. Notably, the sulfonated poly(phenylene) ionomer, Pemion, has recently achieved PEMFC performance on par with Nafion [9, 10]. Despite these advances, durability remains a persistent bottleneck for hydrocarbon ionomer‐based PEMFCs, particularly under the demanding electrochemical conditions in practical operation [11, 12].

Cathode durability is intimately linked to catalyst degradation mechanisms, including Pt dissolution, nanoparticle coalescence, and Pt migration followed by re‐deposition onto larger particles, which collectively leads to the loss of electrochemical active surface area (ECSA) [13, 14]. These processes are intensified by highly acidic environments [15]. While hydrocarbon ionomers typically exhibit higher acid group densities—often 50%–100% greater than PFSA analogues—their weaker sulfonic acid strength (pK_a_ ≈ −1 to −3, compared with ≈ −6.3 for PFSA) results in a higher local pH, potentially suppressing Pt dissolution rates and partially mitigating catalyst degradation [16].

Beyond acidity, ionomer‐catalyst interfacial interactions play a decisive role in electrode durability. In PFSA‐based systems, sulfonate adsorption onto catalyst surfaces enhances ionomer binding, while the perfluoroalkyl backbone exhibits minimal interaction with the catalyst [17]. In contrast, adsorption of hydrocarbon ionomers depends strongly on molecular architecture. Polyaromatic backbones, in particular, exhibit pronounced phenyl adsorption driven by van der Waals interactions involving delocalized π electrons [18]. While such interactions can stabilize catalyst nanoparticles by increasing ionomer binding strength, excessive aromatic adsorption may impede the oxygen reduction reaction (ORR) and increase oxygen transport resistance within the catalyst layer [11, 19, 20]. Moreover, adsorbed aromatic groups of hydrocarbon ionomers are susceptible to electrochemical oxidation at elevated cathode potentials. Unlike PFSA backbones, which remain stable up to ∼3 V, polyaromatic and cyclo‐olefinic backbones can undergo oxidative degradation at potentials above 0.8 V, further limiting PEMFC durability [21, 22, 23].

To address these competing effects, a competitive adsorption strategy has been proposed in which phenyl desorption is promoted through enhanced sulfonate‐catalyst interactions [24]. Increasing the ion exchange capacity (IEC) enhances sulfonate adsorption while simultaneously increasing water uptake (WU), thereby moderating local acidity and stabilizing catalyst particles [25]. However, excessive IEC leads to ionomer swelling, which weakens ionomer‐catalyst interfacial integrity and accelerates degradation [26]. These trade‐offs underscore the need for molecular design strategies that balance ionomer adsorption without compromising transport and interfacial stability under practical fuel cell operating conditions.

Here, we investigate the performance and degradation behavior of hydrocarbon ionomer‐bonded PEMFC cathodes using a commercial sulfonated Diels–Alder poly(phenylene) (Pemion) and a series of custom‐designed sulfonated poly(fluorene) ionomers. By systematically correlating molecular structure, interfacial adsorption behavior, and fuel cell performance, and durability under accelerated potential cycling, and benchmarking against Nafion‐based electrodes, we identify adsorption‐engineered molecular design principles to enable durable, high‐performance hydrocarbon ionomers for next generation PFAS‐free PEMFCs.

## 2 Ionomer Structure and Properties

Figure 1a compares the molecular structures of Nafion, Pemion, and a representative sulfonated poly(fluorene) (F3S55, where “3” denotes the number of fluorine atoms in its side chain and “55” the degree of sulfonation), synthesized via Friedel–Crafts polycondensation and post‐sulfonation (Figures S1–S3). In contrast to Pemion, which features pendant phenyl sulfonic acid groups directly attached to the aromatic backbone, sulfonated poly(fluorene) ionomers incorporate long alkyl sulfonic acid side chains tethered to a rigid fused‐ring backbone. Phenyl sulfonic acid groups are intrinsically more acidic than their alkyl counterparts (pK_a_ ≈ −2.8 vs. −1.9); however, alkyl sulfonic acids are expected to generate a less acidic local environment at the catalyst interface, which may contribute to improved durability [27].

**FIGURE 1:**
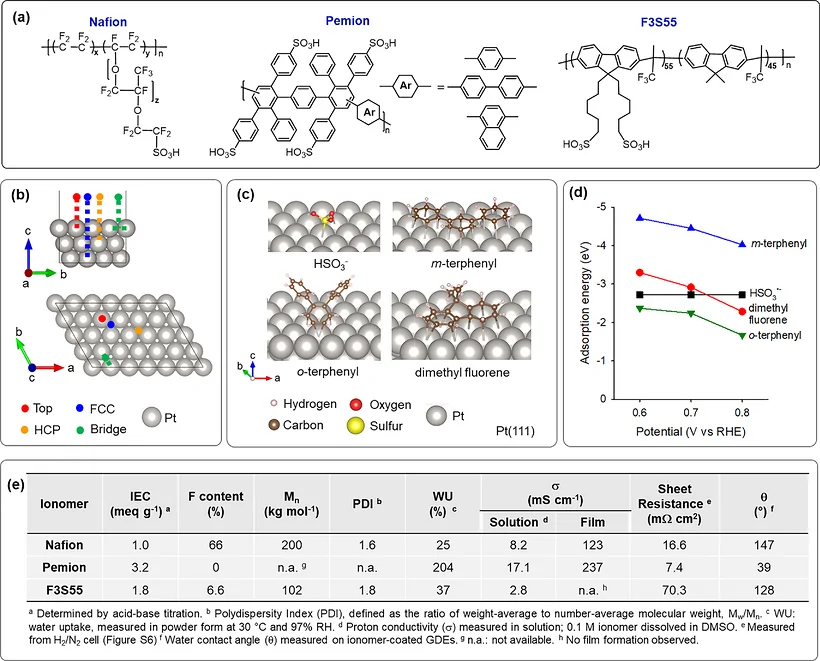
Ionomer structure, interfacial adsorption behavior, and physicochemical properties. (a) Molecular structures of Nafion, Pemion, and F3S55. (b) Schematic illustration of adsorption configurations on the Pt(111) surface, showing the evaluated adsorption sites: top (red), FCC hollow (blue), HCP hollow (orange), and bridge (green). (c) DFT‐calculated adsorption energies of representative ionomer fragments (*m*‐terphenyl, ${\mathrm{HSO}}_{3}^{•-}$, *o*‐terphenyl, and dimethyl fluorene) on Pt(111) as a function of electrode potential. (d) Optimized adsorption geometries of the corresponding fragments on Pt(111), illustrating distinct binding configurations and surface interactions. (e) Summary of key physicochemical properties of Nafion, Pemion, and F3S55 [30], including ion exchange capacity (IEC), fluorine content, number‐average molecular weight (M_n_), polydispersity index (PDI), water uptake (WU), proton conductivity (*σ*) in solution and film, sheet resistance (Figure S6), and water contact angle (*θ*).

To elucidate ionomer‐catalyst interfacial interactions, density functional theory (DFT) calculations were conducted to quantify the adsorption of representative ionomer fragments—hydrogen sulfite (${\mathrm{HSO}}_{3}^{•-}$), *m*‐terphenyl, *o*‐terphenyl, and dimethyl fluorene—on a Pt(111) surface. Four adsorption sites (top, face‐centered cubic (FCC), hexagonal close‐packed (HCP), and bridge) were examined (Figure 1b). For aromatic fragments, both parallel and perpendicular adsorption orientations relative to the Pt surface were considered (Figure S4). Geometry optimization identified the HCP site as the most stable adsorption configuration for ${\mathrm{HSO}}_{3}^{•-}$, corresponding to the strongest adsorption among all species examined. For aromatic fragments, parallel adsorption was consistently favoured over perpendicular orientations (Table S1).

*m*‐Terphenyl adopts a quasi‐planar, extended conformation, with nearly coplanar phenyl rings lying flat on Pt(111), engaging approximately 18 ring carbons in surface contact (average adsorption height, $\bar{z}$ = 2.03 Å) and exhibiting the strongest aromatic adsorption (Figure 1c). In contrast, *o*‐terphenyl retained the non‐coplanar geometry characteristic of its gas‐phase structure [28], with large dihedral angles (68.4° and 74.1°) between the side and central rings. This twisted configuration significantly reduced surface contact (∼4 ring carbons) and resulted in the weakest adsorption ($\bar{z}$ = 3.41 Å). Dimethyl fluorene maintains a nearly planar fused‐ring geometry ($\bar{z}$ = 2.32 Å), interacting with approximately 13 ring carbons and exhibiting intermediate adsorption strength (Figure S5).

At electrode potentials exceeding 0.7 V, the calculated adsorption strength followed the order: *m*‐terphenyl > ${\mathrm{HSO}}_{3}^{•-}$ > dimethyl fluorene > *o*‐terphenyl (Figure 1d). The weak adsorption of *o*‐terphenyl arises from its non‐coplanar configuration, which limits effective interfacial contact and diminishes binding energy. These findings suggest that both Diels–Alder‐derived poly(phenylene)s, containing *o*‐terphenyl motifs, and poly(fluorene)s with rigid fused‐ring backbones exhibit weaker adsorption than linear poly(phenylene) analogues, consistent with prior reports [29]. These calculated adsorption strengths suggest that aromatic adsorption should follow the order m‐terphenyl > fluorene > *o*‐terphenyl, providing a molecular basis for interpreting the electrochemical behavior and durability of the corresponding ionomers discussed below.

Key physicochemical properties of Nafion, Pemion, and F3S55 are summarized in Figure 1e. Pemion exhibits a substantially higher IEC (3.2 meq g^−1^) than Nafion (1.0 meq g^−1^) and F3S55 (1.8 meq g^−1^), enabling higher proton conductivity in both solution and thin films. Consistent with this trend, Pemion‐bonded electrodes display the lowest proton resistance (Figure S6).

A key distinction between Pemion‐ and F3S55‐bonded electrodes lies in their interfacial hydrophobicity. Owing to the presence of fluoroalkyl side chains, F3S55‐bonded electrodes exhibit markedly greater hydrophobicity. Water contact angle measurements reveal a value of 128° for F3S55, approaching that of Nafion (147°), whereas Pemion displays substantially lower hydrophobicity, with a contact angle of 39° (Figure S7). This pronounced difference in wettability is expected to influence ionomer distribution, water management, and interfacial stability within the catalyst layer.

## 3 Ionomer Performance From Half‐Cell Studies

The intrinsic electrochemical behavior of ionomers was first examined using model half‐cell measurements on Nafion thin‐film‐coated Pt/C electrodes in well‐defined model electrolytes. To isolate the role of sulfonic acid chemistry, small‐molecule analogues representing Nafion, Pemion, and sulfonated poly(fluorene) were employed: triflic acid (CF_3_SO_3_H), benzenesulfonic acid (C_6_H_5_SO_3_H), and fluorene sulfonic acid (2,7‐di‐*tert*‐butyl‐9,9‐bis(6‐dihexanesulfonic acid)fluorene) (Figure S8), respectively.

Cyclic voltammograms (CV) recorded in triflic acid closely resemble those obtained in sulfuric acid, yielding comparable ECSAs (∼70 m^2^ g^−1^; Figure 2a). In contrast, hydrogen adsorption is markedly suppressed in benzene sulfonic acid and further reduced in fluorene sulfonic acid (Figure 2b,c), indicating progressively stronger surface blocking by aromatic species through π‐interactions with Pt [31]. At elevated potentials (>0.8 V), both aromatic acids exhibit distinct oxidation currents near 1.0 V, reflecting the electrochemical oxidation of the aromatic groups, while fluorene sulfonic acid shows a lower oxidation current than benzenesulfonic acid, consistent with its improved stability.

**FIGURE 2:**
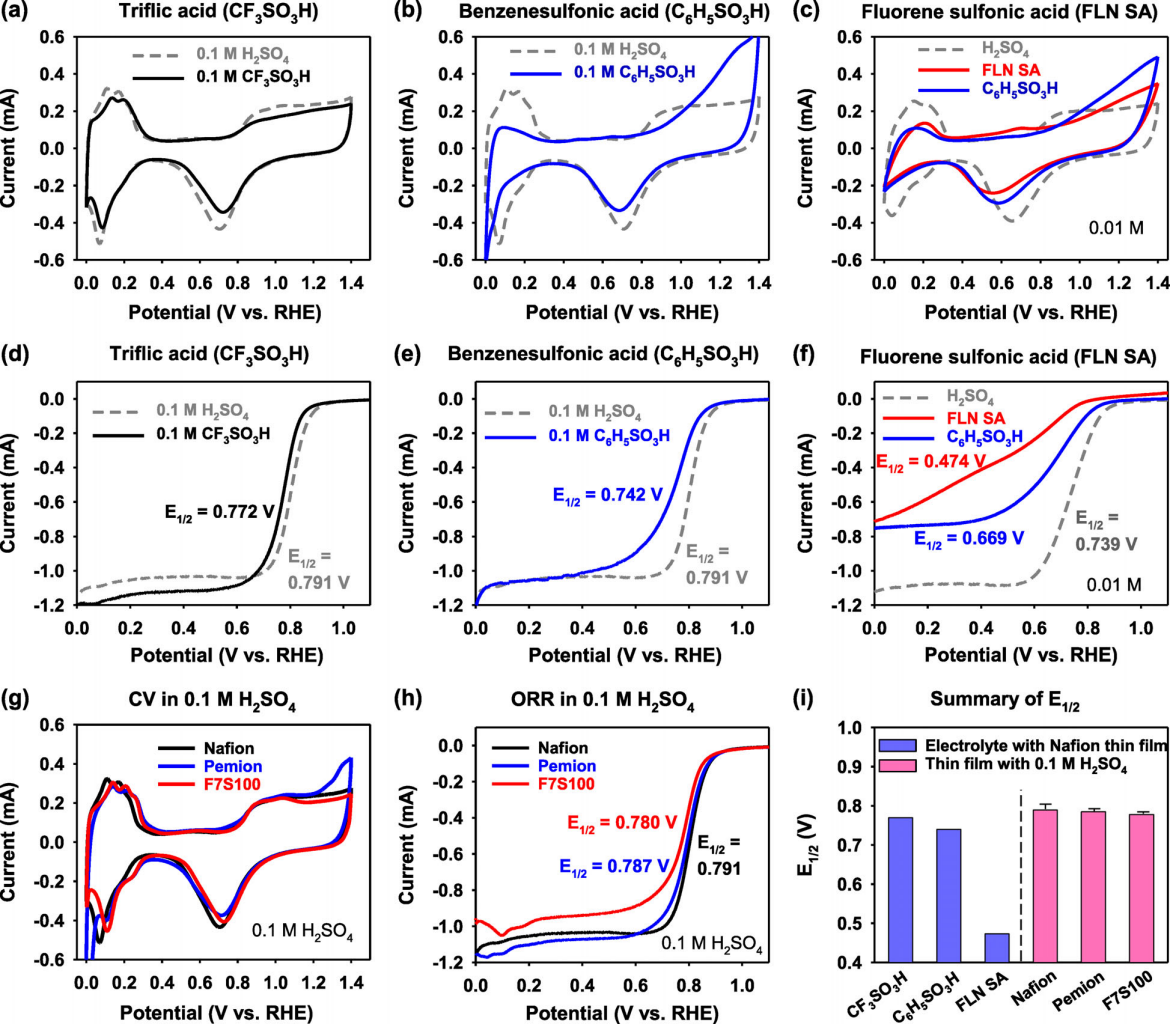
Influence of sulfonic acid chemistry on Pt surface adsorption and ORR kinetics. (a–c) CVs of Nafion‐bonded Pt/C electrodes in model electrolytes containing (a) triflic acid (CF_3_SO_3_H), (b) benzenesulfonic acid (C_6_H_5_SO_3_H), and (c) fluorene sulfonic acid (FLN SA), benchmarked against H_2_SO_4_ and C_6_H_5_SO_3_H as serving as reference electrolytes at identical concentrations. (d–f) Rotating disk electrode (RDE) polarization curves for the ORR in the corresponding electrolytes at (d,e) 0.1 M and (f) 0.01 M. CV and ORR measurements for FLN SA were conducted at 0.01 M due to its low oxygen solubility. (g,h) Electrochemical response of Nafion‐, Pemion‐, and F7S100‐ionomer thin films on Pt/C in 0.1 M H_2_SO_4_, showing (g) CVs and (h) ORR polarization curves. (i) Summary of half‐wave potential (*E*_1/2_) values extracted from model electrolytes and ionomer thin films. All CVs and ORR measurements were conducted at room temperature with a scan rate of 20 mV s^−1^, and 5 mV s^−1^, respectively.

Rotating disk electrode (RDE) measurements (Figure 2d–f) further reveal that ORR activity is largely preserved in triflic acid (*E*_1_/_2_ = 0.772 V) but decreases in benzenesulfonic acid (0.742 V) and is severely suppressed in fluorene sulfonic acid (0.474 V), consistent with strong aromatic adsorption and active‐site blocking. When considered together with the DFT calculations and CV measurements, these results demonstrate that strong adsorption alone does not ensure stable catalyst–ionomer interactions. Under ORR conditions, oxidative transformation of aromatic groups generates more polar, weakly adsorbing species, likely weakening interfacial anchoring and promoting desorption. This dynamic adsorption–oxidation interplay may transiently restore active sites but ultimately weakens catalyst‐ionomer adhesion and promotes interfacial degradation [32].

To probe these effects under catalyst‐layer‐relevant conditions, Pt/C electrodes coated with ionomer thin films were evaluated in sulfuric acid, where sulfonate adsorption from the electrolyte competes with ionomer‐derived species [33]. To approximate the corresponding small‐molecule analoge, a highly sulfonated yet hydrophobic poly(fluorene) (F7S100) was synthesized by increasing the lengh of alkylfluoro side chain from one to three fluorinated carbons (Figure S9 and Table S2). This modification increases the water contact angle from 31° to 84° while reducing the IEC to 2.3 meq g^−1^. In this configuration, ECSAs of all ionomer‐coated electrodes remain comparable (∼70 m^2^ g^−^^1^), indicating effective displacement of aromatic species by mobile sulfonate groups. Notably, pronounced electrochemical oxidation is observed for Pemion‐coated electrodes, whereas negligible oxidation occurs for F7S100, consistent with the greater oxidative stability of the fluorene backbone (Figure 2g).

Despite differences in interfacial chemistry, ORR activity remains high across all ionomer‐coated electrodes (Figure 2h,i), with only minor shifts in half‐wave potential (Nafion: 0.792 V; Pemion: 0.787 V; F7S100: 0.780 V) (Table S3). These observations indicate that competitive sulfonate adsorption mitigates the inhibitory effects of aromatic groups under operating conditions. Consistently, in perchloric acid—where specific anion adsorption is weak—Pemion exhibits pronounced ionomer oxidation and reduced ORR activity, highlighting the critical role of sulfonate‐mediated surface renewal (Figure S10).

Collectively, these half‐cell studies reveal that aromatic adsorption and oxidative stability jointly govern interfacial behavior. While phenyl‐containing ionomers can strongly adsorb on Pt, their susceptibility to oxidation undermines interfacial stability. In contrast, fluorene‐based architectures achieve a more favorable balance between adsorption strength and oxidative stability, enabling more stable catalyst–ionomer interfaces.

## 4 Ionomer Performance in Single Cells

Figure 3 compares CVs and H_2_/air fuel cell performance of membrane electrode assemblies (MEAs) incorporating Nafion, Pemion and F3S55 as cathode ionomers during break‐in. The ionomer‐to‐carbon (I/C) ratio for each catalyst layer was optimized to maximize electrode mass activity (Figure S11). Two carbon supports with distinct pore architectures were examined: non‐porous Vulcan carbon and high‐surface‐area carbon (HSC), the latter featuring a high density of micro‐ and mesopores.

**FIGURE 3:**
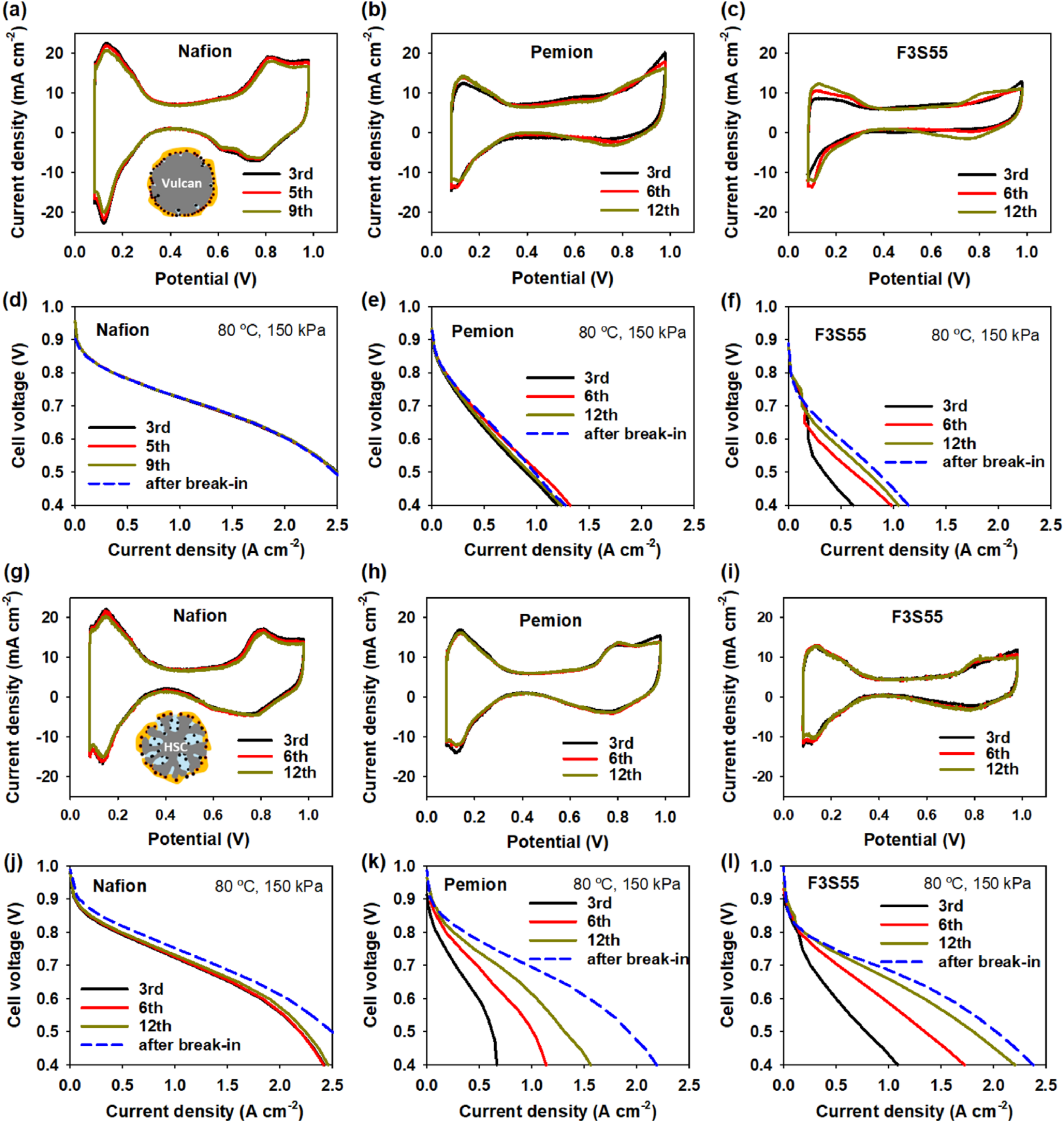
Evolution of electrochemical behavior and fuel cell performance during potential cycling for Nafion‐, Pemion‐, and F3S55‐based cathodes. (a–c) CVs of Vulcan‐supported cathodes during break‐in (Pt loading: Nafion, 0.30 mg cm^−2^, Pemion, 0.30 mg cm^−2^, and F3S55, 0.23 mg cm^−2^, (d–f) Corresponding H_2_/air polarization curves of Vulcan‐supported MEAs measured at 100% RH; Blue dashed lines indicate initial performance after break‐in. (g–i) CVs of HSC supported cathodes during break‐in (Pt loading: Nafion, 0.25 mg cm^−2^, Pemion, 0.26 mg cm^−2^, and F3S55, 0.25 mg cm^−2^), (j–l) Corresponding H_2_/air polarization curves of HSC‐supported MEAs at 100% RH; blue dashed lines indicate initial performance after break‐in. The anode was used as a pseudo‐hydrogen reference electrode for CV measurements.

For Vulcan‐supported cathodes, polyaromatic ionomers (Pemion and F3S55) exhibit two distinct electrochemical signatures in the CV profiles (Figure 3a–c). First, a pronounced reduction in ECSA is observed relative to Nafion‐based electrodes. Following break‐in, the Nafion‐bonded cathode achieves an ECSA of 65.0 m^2^ g_Pt_^−1^, whereas Pemion‐ and F3S55‐based systems display significantly lower values of 37.5 and 44.5 m^2^ g_Pt_^−1^, respectively (Table S4). Second, Pt oxide formation is suppressed near ∼0.7 V, accompanied by the emergence of anodic currents above 0.8 V. Although partial recovery occurs upon extended potential cycling, these features remain incompletely restored after break‐in.

These observations are consistent with half‐cell measurements; however, their persistence in MEAs reflects the absence of a liquid electrolyte, which substantially slows competitive adsorption–desorption dynamics. Notably, relaxation and redistribution of surface‐bound species proceed more readily in F3S55‐bonded electrodes than in Pemion‐based systems, likely due to the greater conformational mobility of alkyl sulfonic acid side chains compared to pendant phenyl sulfonic acid groups.

The corresponding H_2_/air polarization curves for Vulcan‐supported cathodes are presented in Figure 3d–f. Nafion/Vulcan electrodes exhibit rapid break‐in, with performance at the third cycle nearly identical to that at the twelfth cycle, indicating rapid establishment of stable ionomer‐catalyst interfaces. Pemion‐bonded electrodes display similarly short activation periods. In contrast, F3S55‐bonded electrodes undergo a more prolonged activation process, with substantial performance gains between the third and twelfth cycles, attributed to gradual relaxation and reorganization of alkyl sulfonic acid functionalities within the catalyst layer.

Nafion‐bonded cathodes exhibit high performance from the initial cycles, whereas Pemion‐ and F3S55‐bonded cathodes exhibit markedly lower activity after break‐in. At 0.65 V, the Nafion‐bonded cathode achieves 1.72 A cm^−2^, compared to 0.55 and 0.33 A cm^−2^ for Pemion‐ and F3S55‐bonded cathodes, respectively. Kinetic performance at high potentials (>0.8 V) is similarly diminished for polyaromatic ionomers, consistent with mass activity trends (Table S5). Specifically, Nafion achieves a mass activity of 0.23 A mg_Pt_^−1^, exceeding Pemion (0.14 A mg_Pt_^−1^) and F3S55 (0.025 A mg_Pt_^−1^). Despite an approximately sixfold lower intrinsic mass activity than Pemion, the F3S55‐based cathode shows only modest losses in high‐current‐density performance under H_2_/air operation.

Electrochemical impedance analysis (EIS) coupled with equivalent circuit analysis reveals that the mass transport resistance of F3S55 is only ∼20% higher than that of Pemion (Figure S12). This behavior contrasts with half‐cell expectations and is attributed to the intrinsic hydrophobicity of F3S55 and concomitant catalyst layer restructuring during break‐in. These effects likely mitigate gas transport limitations associated with thin ionomer films, thereby enhancing effective oxygen transport under practical operating conditions.

A distinct behavior emerges when HSC is employed as the catalyst support (Figure 3g–i). Owing to the smaller Pt nanoparticle size on HSC (2.2 nm vs. 2.5 nm on Vulcan), an ∼14% increase in ECSA is anticipated. Experimentally, Nafion‐ and F3S55‐based cathodes exhibit modest increases by 8% and 7%, respectively, reflecting particle growth during break‐in (Table S6). In contrast, the Pemion‐based HSC cathode displays a substantially larger increase (∼24%), exceeding size‐based expectations and suggesting partial Pt loss, indicative of weaker ionomer‐Pt interactions. Electrochemical oxidation features support this interpretation: Pemion‐based HSC electrodes display strongly suppressed oxidative currents, whereas F3S55‐based systems display minimal dependence on carbon support, indicating persistent and robust Pt‐ionomer interactions even within the confined pore structure of HSC.

Fuel cell performance under H_2_/O_2_ and H_2_/air conditions highlights the benefits of HSC supports. Under H_2_/O_2_ operation, all systems exhibit enhanced mass activity (Table S7), increasing by ∼2.5‐fold for Nafion‐ and Pemion, and by ∼7.2‐fold for F3S55. Despite this substantial enhancement, the F3S55 cathode (0.18 A mg_Pt_^−1^) remains less active than Nafion (0.54 A mg_Pt_^−1^) and Pemion‐ (0.42 A mg_Pt_^−1^). The pronounced enhancement with HSC is attributed to Pt nanoparticles residing within pores that are largely shielded from ionomer adsorption. The particularly large improvement for F3S55 suggests that performance losses in Vulcan‐supported systems predominantly arise from aromatic adsorption on exposed Pt surfaces and insufficient displacement by alkyl‐linked sulfonate groups.

Figure 3j–l summarizes H_2_/air performance of HSC‐supported cathodes during break‐in. Nafion‐based electrodes again stabilize rapidly, mirroring behavior on Vulcan supports. In contrast, polyaromatic ionomers exhibit extended break‐in periods, reflecting the longer time required for ionomer redistribution within the porous HSC network. This slower equilibration aligns with prior reports of structural reorganization in sulfonated polyaromatic ionomers under Pt oxidation [34], potential cycling [35, 36], and humidity variations [37, 38]. While the driving force—affinity of sulfonic acid groups for hydrophilic Pt oxide surfaces—remains unchanged, the rigid backbones of polyaromatic ionomers impose steric constraints that limit chain mobility and slow structural relaxation.

Such prolonged activation may present challenges for practical implementation, impacting system readiness and operational reliability. Although Pemion‐ and F3S55‐based HSC cathodes display comparable stabilization times, the Pemion‐based system displays more pronounced mass‐transport limitations, likely due to lower hydrophobicity and restricted oxygen transport pathways. This effect likely underlies the superior high‐current‐density performance of F3S55, despite its lower intrinsic oxygen diffusivity relative to Pemion (Figure 2h).

After stabilization, the Nafion‐based HSC cathode delivers performance comparable to its Vulcan‐supported counterpart (1.79 vs. 1.72 A cm^−2^ at 0.65 V). In contrast, both Pemion‐ and F3S55‐based systems exhibit substantial performance gains on HSC. Following break‐in, both polyaromatic ionomers converge to similar high‐current‐density performance, each achieving 1.28 A cm^−2^ at 0.65 V—approximately 28% lower than Nafion. Notably, the performance of the F3S55‐based MEA is comparable to that reported for sulfonated poly(fluorene‐biphenyl) ionomer systems [8].

## 5 Impact of Ionomer on Fuel Cell Durability

Cathode durability was assessed using the Million Mile Fuel Cell Truck (M2FCT) catalyst accelerated stress testing (AST) heavy‐duty protocol, which simulates long‐term fuel cell operation through 90,000 accelerated voltage cycles [39]. This stringent protocol provides a robust benchmark for evaluating ionomer‐driven durability effects in PEMFC cathodes.

To establish molecular design principles governing hydrocarbon ionomer durability, we systematically examined the effects of polymer backbone chemistry, molecular weight, IEC, and side‐chain hydrophobicity using the M2FCT accelerated stress protocol. Direct comparison of Nafion‐, Pemion‐, and F3S55‐bonded HSC cathodes under AST conditions reveals pronounced differences in durability (Figure 4a–c). After 90,000 cycles, the Pemion‐bonded cathode exhibits a 58% loss in current density at 0.65 V, whereas significantly smaller losses are observed for the Nafion‐ and F3S55‐bonded cathodes (14% and 29%, respectively). These results indicate markedly improved durability for the F3S55 ionomer relative to Pemion, despite comparable initial performance.

**FIGURE 4:**
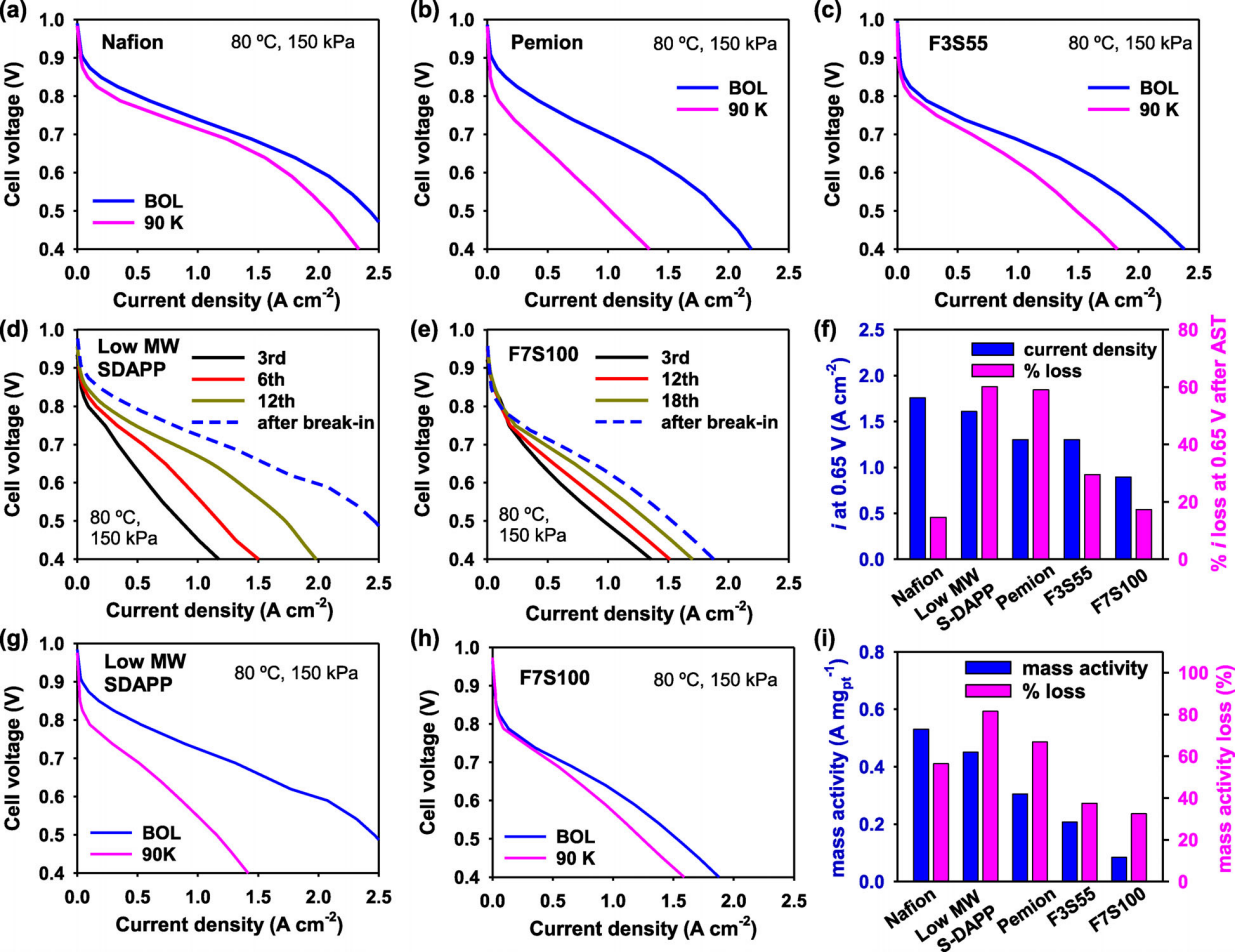
Impact of ionomer chemistry on fuel cell durability and performance degradation under accelerated stress testing. (a–c) H_2_/air polarization curves of HSC‐supported MEAs employing (a) Nafion‐, (b) Pemion‐, (c) F3S55‐based cathodes, comparing beginning‐of‐life (BOL) performance with that after 90K cycles at 80°C and 150 kPa. (d, e) Evolution of polarization behavior of HSC‐supported MEAs during break‐in for (d) low‐molecular weight SDAPP and (e) F7S100‐based cathodes. (f) Summary of current density at 0.65 V and corresponding percentage loss after AST, illustrating the dependence of performance retention on ionomer chemistry. (g, h) Polarization curves of (g) low‐MW SDAPP and (h) F7S100 cathodes comparing BOL and 90K performance. (i) Mass activity and corresponding percentage loss after AST.

The trends in kinetic degradation, evaluated via mass activity, differ from those observed at high current density (Table S8). The Pemion‐bonded cathode experiences a severe mass activity loss of nearly 76%, while the Nafion‐bonded cathode also shows substantial degradation (57%). In contrast, the F3S55‐bonded cathode exhibits a significantly lower mass activity loss of 38% following AST. Importantly, changes in kinetic performance do not correlate directly with ECSA loss. Although all cathodes experience substantial ECSA losses, (62% for Nafion, 65% for Pemion, and 61% for F3S55, Table S9 and Figure S13), and the comparatively small differences in ECSA loss cannot account for the large disparities in performance decay. Collectively, these results underscore the superior durability of F3S55‐bonded cathodes and highlight the critical role of ionomer‐catalyst interfacial chemistry beyond surface area effects.

To elucidate the molecular origins of the enhanced durability associated with sulfonated poly(fluorene) ionomers, a series of systematic structural modifications were investigated. First, a low‐molecular‐weight sulfonated Diels–Alder polyphenylene (Low MW SDAPP, IEC = 3.6 meq g^−1^) was synthesized via post sulfonation of a low‐molecular‐weight precursor (*M*_n_ = 38.7 kg mol^−1^), yielding a polymer chemically analogous to Pemion [40] (Figures S14 and S15; Table S2). This material was designed to assess whether reduced molecular weight impacts access to Pt nanoparticles within pores and enhances ionomer dispersion within the catalyst layer.

The CVs of Low‐MW SDAPP‐bonded cathodes during break‐in closely resemble those of Pemion‐bonded electrodes. (Figure 4d and Figure S16). Although the initial high‐current‐density performance is substantially improved, reaching 1.58 A cm^−2^ at 0.65 V—suggesting that the reduced molecular weight promotes a more favorable catalyst‐layer morphology for oxygen transport (Figure S17)—severe degradation is observed following AST.

Specifically, the Low‐MW SDAPP‐bonded cathode exhibits a 59% loss in current density at 0.65 V after 90,000 cycles (Figure 4g), accompanied by pronounced reductions in mass activity (83%) and ECSA (62%) (Tables S8 and S9; Figure S18). These degradation levels are comparable to those observed for Pemion‐bonded cathodes. These resurts indicate that reducing molecular weight alone is insufficient to improve the durability of phenylene‐based ionomers. This instability is likely attributable to the low adsorption energy of *o*‐terphenyl units within the polymer backbone.

In contrast, targeted molecular engineering of the poly(fluorene) ionomer framework yields substantial improvements in durability. To enhance sulfonate adsorption and thereby increase durability, the degree of sulfonation of F3S55 was increased from 55% to 100%, yielding F3S100 (IEC = 2.8 meq g^−1^ compared with 1.8 meq g^−1^ of F3S55) (Figure S19). While this modification improved durability, it reduced mass activity and high‐current‐density performance, likely due to reduced hydrophobicity and increased oxygen transport resistance (Tables S2 and S8; Figure S20).

The durability of F3S100‐bonded cathodes was further examined as a function of ionomer molecular weight (*M*_n_ = 14, 26, and 49 kg mol^−1^). While initial performance increases modestly with decreasing molecular weight (0.85, 0.80, and 0.72 A cm^−2^ at 0.65 V, respectively; Figure S21), all F3S100‐bonded cathodes exhibited substantially improved durability compared to F3S55 (<20% loss in current density at 0.65 V after 90,000 cycles) (Figure 4). Mass activity and ECSA losses are likewise limited to 27% and 32%, respectively (Tables S8 and S9; Figure S22), representing a substantial improvement over F3S55‐bonded cathodes (38% and 61%). These results demonstrate that the sulfonated poly(fluorene) backbone intrinsically promotes durability, largely independent of molecular weight.

The break‐in behavior of highly sulfonated, hydrophobic poly(fluorene) F7S100‐based cathodes resembles that of F3S55 (Figure 4e). Although F7S100‐bonded cathodes showed improved high‐current‐density performance (0.88 A cm^−2^ at 0.65 V) compared to F3S100‐bonded cathodes (∼0.80 A cm^−2^) likely due to the enhanced hydrophobicity, their performance remained lower than that of F3S55‐bonded cathodes (1.28 A cm^−2^), consistent with the overall hydrophobicity trend governed by fluoroalkyl chain length and IEC (F3S100 < F7S100 < F3S55). However, F7S100 exhibits exceptional durability (Figure 4h); after 90,000 potential cycles, the current density loss at 0.65 V is limited to only 17% (Figure 4f), the lowest among all MEAs evaluated. Mass activity and ECSA losses are similarly minimized to 33% and 39%, respectively (Table S8 and Figure S23).

Figure 4f compares the initial current density and the percentage performance loss at 0.65 V after AST for all MEAs studied. Cathodes bonded with sulfonated Diels–Alder poly(phenylene) ionomers (Pemion and Low MW SDAPP) exhibit relatively high initial performance but suffer severe degradation. In contrast, sulfonated poly(fluorene) ionomers (F3S55 and F7S100) achieve comparable initial performance with dramatically reduced performance decay. Notably, F3S55 retains an initial performance similar to Pemion while reducing performance loss by approximately 50%.

Figure 4i further correlates the initial mass activity with mass activity loss after AST. Among hydrocarbon ionomer‐bonded cathodes, higher initial mass activity is systematically associated with greater degradation. These results reveal a fundamental tradeoff between initial activity and long‐term durability: stronger ionomer‐catalyst interactions suppress initial kinetics but substantially stabilize the catalyst layer during long‐term operation. Notably, both sulfonated poly(fluorene) ionomers exhibit lower mass activity loss than Nafion‐bonded cathodes, underscoring the promise of adsorption‐engineered hydrocarbon ionomers for durable, PFAS‐free PEMFCs.

## 6 Mechanism for Fuel Cell Durability Enhancement

The origin of enhanced fuel cell durability in sulfonated poly(fluorene) ionomers was investigated using cross‐sectional high‐angle annular dark‐field scanning transmission electron microscopy (HAADF‐STEM) coupled with energy‐dispersive x‐ray spectroscopy (EDS). Pt loss from the cathode to the membrane after 90,000 potential‐cycling ASTs was quantified by comparing catalyst layers before and after cycling (Figure 5a–f and Figure S24). The reported Pt loss values reflect redistribution and redeposition within the membrane and are therefore interpreted as qualitative indicators of dissolution trends rather than absolute measures of total Pt loss.

**FIGURE 5:**
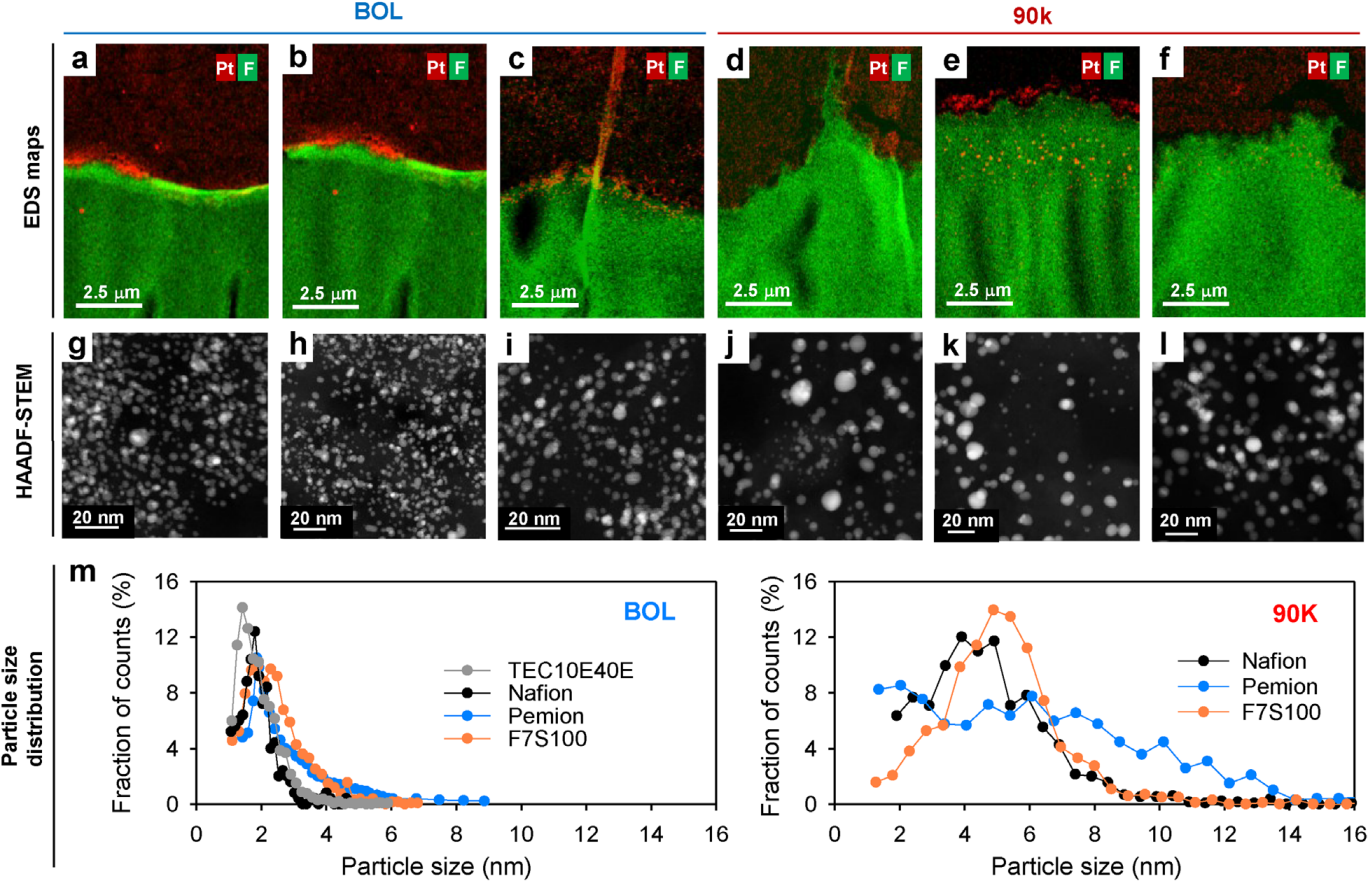
Structural and nanoscale evolution of cathode catalyst layers after accelerated stress testing and implications for durability. (a–f) Cross‐sectional HAADF‐STEM elemental map (Pt: magenta; F: green) of Nafion‐, Pemion‐ and F7S100‐bonded cathodes at beginning‐of‐life (BOL, a–c) and after 90,000 potential cycles (90K, d–f). (g–l) Representative high‐resolution HAADF‐STEM images of Pt nanoparticles in Nafion‐, Pemion‐, and F7S100‐bonded cathodes at BOL (g–i) and after 90K cycling (j–l), (m) Corresponding particle size distributions for Nafion‐, Pemion‐, and F7S100‐bonded cathodes at BOL and after 90K cycling.

Nafion‐based cathodes exhibited moderate Pt loss (∼16%), whereas Pemion‐based cathodes show severe degradation accompanied by extensive Pt redistribution into the membrane. Image analysis indicates that ∼70% of the initial Pt inventory was lost from the Pemion electrode. In contrast, the F7S100‐bonded cathode retained most of its Pt, with only ∼8% loss, and redistribution largely confined to the membrane‐electrode interface.

Post‐AST HAADF‐STEM analysis reveals distinct nanoparticle evolution pathways (Figure 5g–l). The Pemion‐based cathode exhibits pronounced particle coarsening accompanied by a substantial reduction in nanoparticle population, resulting in a broad size distribution with a significant fraction of particles exceeding 9 nm (Figure 5m). In contrast, Nafion‐ and F7S100‐based cathodes retain unimodal size distributions. Nafion retains a larger fraction of particles smaller than 4 nm, likely stabilized within microporous regions of the carbon support.

The Pemion electrode also retains a population of sub‐2 nm nanoparticles, suggesting that particles confined within carbon pores remain relatively stable, while larger, ionomer‐exposed particles preferentially dissolve and migrate into the membrane. This apparant is attributed to confinement within mesopores, where limited ionomer accessibility creates a locally less acidic environment.

In the F7S100 system, Pt particle sizes remained predominantly within the 2–7 nm range after AST, consistent with suppressed particle growth and stabilization of larger nanoparticles through strong ionomer‐catalyst interactions.

These structural trends translate directly into electrochemical performance and durability. The low mass activity of the Pemion‐based cathode is primarily driven by extensive Pt loss rather than particle coarsening. This degradation originates from unstable catalyst‐ionomer interactions where competitive adsorption between phenyl and sulfonate groups weakens interfacial binding, while oxidative degradation of the aromatic backbone produces more polar, weakly adsorbing oxidation products. In contrast, Nafion maintains catalyst integrity despite weak backbone interactions, owing to strong sulfonate‐Pt binding and the high oxidative stability of its perfluorinated framework. However, its overall interaction strength remains lower than that of F7S100, which benefits from additional backbone‐mediated interactions.

Overall, catalyst durability is dictated by the combined effects of ionomer‐Pt interaction strength and oxidative stability (Figure 6). Nafion achieves stability through chemical robustness and persistent sulfonate adsorption, whereas Pemion undergoes electrochemical oxidation under ORR conditions (e.g., via •OH/H_2_O_2_ attack), leading to ionomer decomposition, Pt detachment, and extensive dissolution. In contrast, F7S100 provides strong and stable interfacial anchoring while resisting oxidative degradation, thereby suppressing Pt migration and coarsening. Although these stronger ionomer‐catalyst interactions slightly compromise initial kinetics, it enables substantially improved catalyst stability and long‐term electrode durability. Collectively, these results establish adsorption engineering as an effective molecular design strategy for developing durable, PFAS‐free hydrocarbon ionomers for PEMFC cathodes.

**FIGURE 6:**
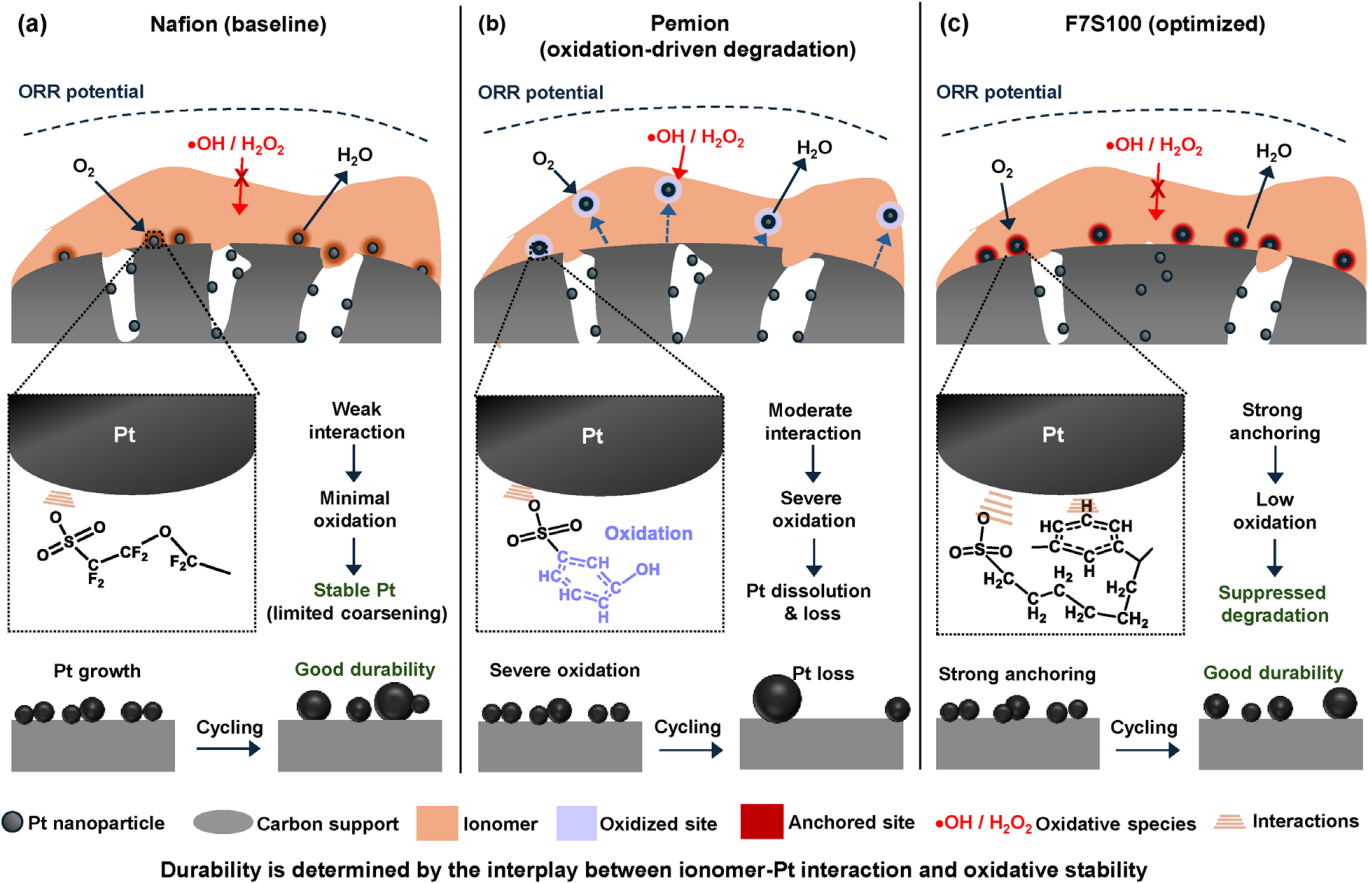
Ionomer‐Pt interaction and oxidative stability jointly govern catalyst durability under ORR conditions. Schematic illustration of ionomer‐Pt interfacial behavior and its impact on catalyst degradation for (a) Nafion, (b) Pemion, and (c) F7S100 under ORR potentials.

## 7 Conclusions

To date, the development of hydrocarbon ionomeric binders for fuel cell cathodes has largely focused on maximizing electrochemical performance. Here, we demonstrate that enhanced kinetic performance can be achieved by attenuating ionomer–catalyst interactions; however, weakening of these interactions may compromise electrode durability due to insufficient interfacial binding. Importantly, we show that oxidative stability of ionomers is an equally critical, yet often overlooked, parameter governing long‐term catalyst integrity. Under ORR conditions, oxidative degradation of hydrocarbon backbones (e.g., via •OH/H_2_O_2_ attack) can generate weakly adsorbing species, accelerate ionomer decomposition, and promote Pt detachment and dissolution.

By employing a linear poly(fluorene) backbone, we establish an effective balance between interaction strength and oxidative resilience, achieving outstanding electrode stability while maintaining competitive performance near 0.65 V, despite the intrinsically lower mass activity of poly(fluorene)‐based systems. These results provide direct experimental validation of a fundamental performance—durability trade‐off in hydrocarbon ionomer‐based cathodes, governed by the interplay between ionomer—Pt interaction strength and resistance to oxidative degradation.

More broadly, our findings highlight that the strong phenyl adsorption characteristic of polyaromatic ionomers, while detrimental to reaction kinetics, can enhance catalyst stabilization. However, without sufficient oxidative robustness, such interactions alone are insufficient to prevent long‐term degradation. Thus, mitigating ionomer oxidation while tuning adsorption strength remains a central challenge in hydrocarbon ionomer design.

Our combined DFT and experimental results suggest that this trade‐off can be addressed through targeted molecular and structural design. Alkyl‐tethered sulfonic acid groups enable co‐adsorption with aromatic backbones while improving conformational flexibility, and fluorene‐based fused‐ring structures provide intermediate adsorption strength with enhanced oxidative stability. In parallel, porous carbon support structure can spatially restrict ionomer coverage on Pt surfaces, reducing kinetic penalties while preserving stabilization.

Together, these results establish adsorption engineering as an effective molecular design strategy for durable, PFAS‐free hydrocarbon ionomers.Continued advances in ionomer molecular architecture will be essential for enabling hydrocarbon systems to achieve performance and durability comparable to—or surpassing—state‐of‐the‐art PFSA materials.

## 8 Methods

### 8.1 Materials

Fluorene, 1,6‐dibromohexane, sodium hydroxide (NaOH), sodium chloride (NaCl), 9,9‐dimethylfluorene, trifluoromethanesulfonic acid, methanol, and acetone were purchased from Fisher Chemical. Tetrabutylammonium iodide, anhydrous dichloromethane (DCM), hydrogen chloride (HCl), magnesium sulfate, silica gel, hexane (mixture of isomers), 1,1,1‐trifluoroacetone, anhydrous *N,N*‐dimethylacetamide (DMAc), anhydrous tetrahydrofuran (THF), 3‐chloroperoxybenzoic acid (mCPBA), 2,7‐di‐*tert*‐butylfluorene, anhydrous *N,N‐*dimethylformamide (DMF), and anhydrous dimethyl sulfoxide (DMSO), 1‐propanol, and isopropyl alcohol were purchased from Sigma‐Aldrich and used as received without further purification.

3,3,4,4,4‐Pentafluorobutan‐2‐one and 3,3,4,4,5,5,5‐heptafluoropentan‐2‐one were obtained from Manchester Organics. Sodium sulfite was acquired from Alfa Aesar. PF1‐HLF8‐15‐X (Pemion) membranes was obtained from Ionomr Innovations Inc., while Nafion D2020 and Nafion NC700 membranes were purchased from DuPont and Chemours, respectively. Commercial Pt/C catalysts, including TEC10V20E (20% Pt), TEC10E40E (40% Pt), and TEC10E50E (50% Pt) were purchased from TANAKA Precious Metals (TKK).

### 8.2 Synthesis of Br‐Containing Fluorene Monomer

9,9‐Bis(6‐bromohexyl)fluorene was synthesized following a modified literature procedure [40]. Fluorene (33 mmol), tetrabutylammonium iodide (3.3 mmol), and 50% aqueous NaOH (96 mL) were added to a 500 mL Schlenk flask under a nitrogen atmosphere. The reaction mixture was degassed by three freeze‐pump‐thaw cycles. Degassed 1,6‐dibromohexane (230 mmol) was then added under nitrogen, and the mixture was heated to 70 °C and stirred overnight.

After cooling to room temperature, the reaction mixture was extracted with DCM. The combined organic layers were sequentially washed with water, 1 M HCl, water, and brine, then dried over anhadrous MgSO_4_. Excess 1,6‐dibromohexane was removed by vacuum distillation. The crude product was purified by column chromatography (SiO_2_, hexane/DCM 19:1, v/v) to afford 9,9‐bis(6‐bromohexyl)fluorene as a white solid in 60% yield.

### 8.3 Synthesis of Sulfonated Poly(fluorene) Ionomers

Sulfonated polyfluorene was synthesized via a three‐step polymerization and post‐functionalization procedure. In a typical synthesis, 9,9‐bis(6‐bromohexyl)fluorene (1.1 mmol), 9,9‐dimethylfluorene (0.92 mmol), and partially fluorinated ketone monomer (3.1 mmol; 1,1,1‐trifluoroacetone, 3,3,4,4,4‐pentafluorobutan‐2‐one, or 3,3,4,4,5,5,5‐heptafluoropentan‐2‐one) were charged into a 25 mL round‐bottom flask under a nitrogen atmosphere. DCM (13 mL) was added, and the mixture was stirred until complete dissolution. The solution was cooled in an ice bath, after which trifluoromethanesulfonic acid (2.3 mL) was added dropwise. The reaction mixture was maintained at 0°C for 15 min and then allowed to warm to room temperature. The polymerization was continued for an additional 15 min (for 1,1,1‐trifluoroacetone) or up to 24 h (for 3,3,4,4,5,5,5‐heptafluoropentan‐2‐one), depending on the reactivity of the fluorinated ketone monomer.

Upon completion, the reaction mixture was precipitated into methanol (100 mL). The resulting white precipitate was collected by filtration, dissolved in THF, and reprecipitated in methanol. The brominated fluorene polymer (F*x*Br*y*) was isolated by filtration and dried under vacuum, yielding the product in 93%.

For thioacetate substitution, F*x*Br*y* (0.54 mmol) was dissolved in DMAc (3 wt%) and potassium thioacetate (2.7 mmol) was added. The mixture was stirred at 50°C for 24 h. After cooling, the reaction solution was precipitated into methanol, and the resulting polymer (F*x*SAc*y*) was collected, washed thoroughly with methanol, and dried under vacuum (78% yield).

Oxidation to the sulfonated polymer was carried out by dissolving F*x*SAc*y* (0.44 mmol) dissolved in DMAc (3 wt%) and cooled to 0°C. mCPBA (2.2 mmol) was added portion‐wise, and the reaction was maintained in an ice bath for 2 h. The reaction mixture was then precipitated into 1 M NaCl solution and filtered. The collected solid was dried, dissolved in DMSO, and reprecipitated into ethyl acetate. After washing repeatedly with ethyl acetate, the final sulfonated poly(fluorene) (F*x*S*y*) was obtained as a white powder dried under vacuum (60% yield).

### 8.4 Synthesis of butyl‐2,7‐di‐*tert*‐butyl‐9,9‐bis(6‐dihexanesulfonic acid)fluorene

2,7‐Di‐*tert*‐butyl‐9,9‐bis(6‐bromohexyl)fluorene was synthesized following a modified literature procedure [41]. Briefly, 2,7‐di‐*tert*‐butylfluorene (36 mmol), tetrabutylammonium iodide (3.6 mmol), and 50 wt% aqueous NaOH (129 mL) were charged into a 500 mL Schlenk flask under a nitrogen atmosphere. The mixture was degassed by three freeze‐pump‐thaw cycles, after which degassed 1,6‐dibromohexane (54 mmol) was added under nitrogen. The reaction mixture was heated to 70°C and stirred for 72 h.

After cooling to room temperature, the reaction mixture was extracted with DCM. The organic phase was sequentially washed with water, 1 M HCl, water, and brine, then dried over MgSO_4_. Excess 1,6‐dibromohexane was removed by vacuum distillation, and the crude product was purified by column chromatography (SiO_2_, hexane/DCM 19:1, v/v) to afford 2,7‐di‐*tert*‐butyl‐9,9‐bis(6‐bromohexyl)fluorene in 12% yield.

Sulfonation of the small molecule was carried out by dissolving 2,7‐di‐*tert‐*butyl‐9,9‐bis(6‐bromohexyl)fluorene (1.5 mmol) in DMF (10 mL) under nitrogen. Sodium sulfite (7.3 mmol), dissolved in deionized water (6.7 mL), was added dropwise, and the reaction mixture was refluxed at 140°C for 120 h. Upon completion, the reaction was cooled to room temperature, and the DMF/water solvent mixture was removed under reduced pressure. The residue was dissolved in methanol, and insoluble impurities were removed by filtration. The filtrate was recrystallized from methanol three times, followed by washing with cold acetone. The resulting white solid was collected by filtration and dried under vacuum to yield 2,7‐di‐*tert*‐butyl‐9,9‐bis(6‐dihexanesulfonic acid)fluorene in 57% yield. The purity of the compound was determined to be 87% by ^1^H NMR spectroscopy (Figure S8).

### 8.5 Synthesis of Sulfonated Diels–Alder Poly(phenylene)s

Sulfonated Diels–Alder poly(phenylene)s were synthesized following previously reported procedures [42, 43]. The molecular weight of the starting Diels–Alder poly(phenylene) was adjusted through stoichiometric imbalance of the bis(tetracyclone) and 1,4‐bis(ethynyl) benzene monomer targeted a molecular average weight of 30K. Diels–Alder poly(phenylene) (7 g, 9.2 mmol) was dissolved in 500 mL of DCM in a 1 L three neck round‐bottom flask equipped with a nitrogen inlet, glass stopper, and 60 mL addition funnel. After complete dissolution, the solution was cooled in an ice bath for 30 min. Trimethylsilyl chlorosulfonate (16 mL, 0.1 mol), along with 20 mL of DCM, was then added dropwise. The reaction mixture gradually darkened from bright yellow to brown and was stirred for at least 10 h, during which a dark brown fibrous solid precipitated.

### 8.6 IEC Measurements

The IEC of the polymers was determined by equilibrating polymer powder in 20 mL of 1 M aqueous NaCl solution for 24 h under stirring to fully exchange proton (H^+^) to sodium ions (Na^+^). The IEC was then quantified using a conventional acid‐base titration method. Titration was carried out with a standardized 0.01 M aqueous NaOH solution, using 5 wt.% phenolphthalein in ethanol as the indicator.

The IEC was calculated according to Equation (1):

(1) $$\mathrm{IEC}\;\;\left(\mathrm{meq}\;g^{-1}\right)=\frac{V\;\times C}{w}\;$$

where *V* is the volume of NaOH solution consumed during titration (*L*), *C* is the molar concentration of the NaOH solution (*mol L^−1^*); and *w* is the mass of the dried polymer sample (g).

### 8.7 Chemical Structure Analysis by NMR

Nuclear magnetic spectroscopy (NMR) samples were prepared by dissolving the synthesized compounds in deuterated chloroform (CDCl_3_), deuterated dimethyl sulfoxide (DMSO‐d_6_), deuterated methanol (MeOD), or deuterium oxide (D_2_O), as appropriate. ^1^H and ^19^F NMR spectra were acquired using a Bruker 400 NMR spectrometer.

### 8.8 GPC Measurements

Gel permeation chromatography (GPC) (Agilent Technologies 1260 Infinity II system (G7800A 1260 GPC/SEC MDS, G7116A 1260 MCT, and G7129A 1269 Vial Sampler) to determine the number‐average molecular weight (M_n_) and polydispersity index (PDI). Separations were carried out using PL1117‐6800 Polargel‐M (300 × 7.5 mm) with THF (HPLC grade) as the eluent at a flow rate of 1.0 mL min^−1^. Molecular weight calibration was conducted using polystyrene standards.

### 8.9 Water Uptake Measurements

Water uptake (WU) was determined by placing dried polymer powder in a vial and equilibrating it in a temperature‐ and humidity‐controlled chamber at 97% relative humidity and 30°C for 24 h. WU of Nafion and Pemion was determined in their membrane form. Membranes were soaked in 1 M H_2_SO_4_ at 80°C for 1 h, followed by rinsing with Milli‐Q water for 24 h. Excess water on the membrane surface was gently removed with Kimwipes, and then wet weight was recorded. Membranes were dried at 80°C overnight to obtain dry weight. The WU was calculated using Equation (2):

(2) $$\mathrm{Water}\;\mathrm{Uptake}\;\;\left(\%\right)=\frac{w_{\mathrm{wet}}-\;w_{\mathrm{dry}}}{w_{\mathrm{dry}}}\times 100\;\left(\%\right)\;$$

where *w*_wet_ and *w*_dry_ denote the weights of the hydrated and dry samples, respectively.

### 8.10 Impedance Measurements

Solution conductivity (*σ*) was measured using AC impedance spectroscopy with a two‐probe configuration at 80°C and 97% RH. A Solartron‐1260 impedance/gain‐phase analyzer was used to recorded impedance spectra over a frequency range of 1 to 1 000 000 Hz. The ionomer solution in the H^+^ form was prepared by dissolving the ionomer at a concentration of 0.1 M in DMSO. The resulting solution was loaded into a conductivity measurement cell and equilibrated at 80°C prior to measurement. In‐plane proton conductivity of membrane was measured at 80°C in water. Membranes were placed between platinum‐coated electrode and equilibrated for 1 h. The measured impedance of solution and membrane was converted to proton conductivity using Equation (3):

(3) $$\mathrm{Proton}\;\mathrm{Conductivity}\;\;\left(S\;cm^{-1}\right)=\sigma =\frac{1}{R}\;\times \frac{L}{A}\;$$

where *R* is the measured impedance value (Ω), *L* is the distance between the electrodes, and *A* is the cross‐sectional area (cm^2^) of the ion‐exchange membrane used in the measurement.

### 8.11 Contact Angle Measurements

Contact angle measurements were performed using a Ramé‐Hart Model 500 goniometer. Gas diffusion electrodes (GDEs) were fabricated by spray‐coating an ink containing Pt/C catalyst and ionomer onto an Avcarb MB‐30 gas diffusion layer. The contact angle was measured 60 s after depositing a water droplet onto the GDE surface.

### 8.12 Computational Details

To quantitatively evaluate the adsorption behavior of hydrogen sulfite radical anion, *m*‐terphenyl, *o*‐terphenyl, and dimethyl fluorene on the Pt(111) surface, spin‐polarized density functional theory (DFT) calculations were performed using the Vienna Ab Initio Simulation Package (VASP) [44, 45]. The interactions between core and valence electrons were described by the projector augmented wave (PAW) method [46, 47], while the exchange‐correlation potential was treated within the generalized gradient approximation (GGA) framework, utilizing the Perdew–Burke–Ernzerhof (PBE) functional [48]. To incorporate van der Waals interactions, the DFT‐D3 dispersion correction was applied [49]. A plane‐wave energy cutoff of 400 eV was adopted for all calculations, and the Brillouin zone was sampled using a Monkhorst‐Pack *k*‐point grid of 1 × 2 × 1 [50]. Methfessel–Paxton smearing was used for electronic occupations [51]. To prevent interlayer interactions, a vacuum layer of 15 Å was introduced along the *c*‐axis. The slab model for the Pt(111) surface consisted of a 3 × 2 × 1 supercell with a three‐layer slab configuration [52, 53, 54]. The bottom two layers of the Pt slab were fixed, while the top layer was fully relaxed to achieve structural optimization. A dipole correction was systematically applied to eliminate electrostatic interactions arising from periodic boundary conditions [55]. Convergence thresholds were set at 10^−5 ^eV for electronic energy and 0.01 eV/Å for atomic forces. The adsorption energy (*E_ads_*) of the adsorbed molecule was calculated using Equation (4) [54, 56]:

(4) $$E_{\mathrm{ads}}=E_{\mathrm{total}}\;-E_{\mathrm{molecule}}-E_{\mathrm{sub}}\;$$

where *E*_total_ is the total energy of the molecule adsorbed on the Pt(111) surface, *E*_molecule_ is the total energy of molecules in vacuum, and *E*_sub_ corresponds to the energy of the clean Pt(111) surface. The average adsorption height ($\bar{z}$) is the mean perpendicular distance of all ring carbons to the least‐squares plane of the topmost Pt layer [57, 58]. And the number of ring carbons in close contact was defined as the number of ring carbons having a C–Pt distance less than 3.4 Å [59].

### 8.13 Half‐Cell Measurement

Catalyst inks were prepared by dispersing 5 mg of commercial Pt/C (TEC10E50E, TKK) with 60 µL of 5 wt% ionomer solution—Nafion (Ion Power), 5 wt% F7S100, or Pemion—in 760 µL of a mixed solvent consisting of 85 wt% 1‐propanol and 15 wt% deionized water. The resulting suspensions were sonicated for 1 h to ensure homogeneous dispersion.

For electrochemical measurements, 5 µL of the catalyst ink was drop‐cast onto a glassy carbon rotating disk electrode (RDE: 5.0 mm diameter, geometric area = 0.196 cm^2^). CV and ORR measurements were conducted using an SP‐150e Potentiostat (BioLogic) in a conventional three‐electrode electrochemical cell. An Ag/AgCl electrode (3.0 M NaCl) and a Pt wire were employed as the reference and counter electrodes, respectively.

Prior to CV measurements, the electrolyte was purged with nitrogen for 30 min to remove dissolved oxygen‐free environment. CVs were recorded between 0.0 and 1.4 V versus the reversible hydrogen electrode (RHE) at a scan rate of 20 mV s^−1^ under static conditions (0 rpm) at room temperature. For ORR activity evaluation, the electrolyte was saturated with oxygen for 30 min, followed by linear sweep voltammetry (LSV) measurements conducted from 1.2 to 0.0 V versus RHE at a scan rate of 5 mV s^−1^ and a rotation rate of 1600 rpm.

All measured potentials were initially referenced to the Ag/AgCl electrode and subsequently converted to the RHE scale.

### 8.14 Polymer Dispersions

Ionomer solutions of F7S100 and F3S55 were prepared by dissolving the polymers in ethanol at a concentration of 5 wt%. Dispersion of Pemion (5 wt%), F3S100(*M*_n_ = 14, 26, and 49 kg mol^−1^), and Low MW SDAPP (5 wt%) were prepared in a 1:1 (w/w) mixture of 1‐propanol and water and heated at 80°C overnight to ensure complete dispersion.

### 8.15 MEA Fabrication

Catalyst‐coated membranes (CCMs) were fabricated via spray deposition onto Nafion NC700 membranes, using a high‐surface‐area carbon‐supported catalyst (TEC10E40E) and a low‐surface‐area carbon‐supported catalyst (TEC10V20E). Catalyst inks were formulated by dispersing the catalyst powders with ionomer solutions, including Nafion D2020 (DuPont), Pemion (Ionomer Innovations), and custom‐prepared ionomers (F7S100, F3S55, F3S100, and Low MW SDAPP), in a mixed solvent of isopropanol and deionized water (9.6:1, v/v).

For the high‐surface‐area carbon‐supported catalysts, the I/C weight ratio was fixed at 0.58 for Pemion, F7S100, F3S55, F3S100, and Low MW SDAPP, while a higher I/C ratio of 0.9 was employed for Nafion‐based inks with TEC10E40E. In contrast, an I/C ratio of 0.5 was used for low‐surface‐area carbon‐supported catalysts employing TEC10V20E. The inks were sonicated for 25 min to ensure homogeneous dispersion prior to spray coating.

Cathode catalyst layers were deposited using a Sono‐Tek spray‐coating system to yield a 5 cm^2^ active area with a platinum loading of 0.25 mg_Pt_ cm^−2^. The Pt loading of low‐surface‐area carbon‐supported catalysts (TEC10V20E) was maintained at 0.3 mg_Pt_ cm^−2^. Anode catalyst layers were similarly prepared using TEC10E40E, with Pt loadings in the range of 0.05–0.1 mg_Pt_ cm^−2^. The Pt loading was confirmed by x‐ray fluorescence spectroscopy (XRF, ARL QUANT'X EDXRF).

MEAs were assembled by sandwiching the CCM between anode and cathode gas diffusion layers (SGL 22BB, SGL Carbon), with polyurethane gaskets (Poron, Rogers) used to ensure proper sealing and compression.

### 8.16 Break‐In and Fuel Cell Performance Evaluation

MEAs were evaluated in a single‐cell configuration using a Scribner 850 fuel cell test system equipped with differential cell hardware. Cell performance was assessed through mass activity, polarization behavior, and ECSA measurements.

All MEAs were preconditioned according to the M2FCT protocol. The cells were initially heated to 80 °C and held at 0 V for 30 min while supplying H_2_ and air at flow rates of 1000 and 3000 standard cubic centimeters per minute (sccm), respectively, under an absolute pressure of 150 kPa and 100% relative humidity.

Subsequent conditioning was performed through a sequence of potentiostatic holds at 0.6 V for 2 min, followed by 0 V for 10 min, and 0.6 V for an additional 5 min. The cells were then subjected to repeated voltage cycling between 0.4 and 0.95 V, with each cycle lasting 4 min, for a total of nine to twenty‐seven cycles (Table S10).

Throughout the preconditioning procedure, current responses were continuously recorded at 10s intervals. To obtain the H_2_/O_2_ polarization curve, the cell was subjected to repeated voltage cycling between 0.4 and 0.95 V, with each cycle lasting 4 min, for one additional cycle under H_2_/O_2_ oat flow rate of 1000 and 3000 sccm.

Single‐cell fuel cell performance was evaluated using H_2_ (1000 sccm) at the anode and either air (3000 sccm) at the cathode, under fully humidified conditions (100% RH). All measurements were conducted at 80°C with an absolute pressure of 150 kPa applied to both electrodes. Mass activity was determined by holding the cell at a constant potential of 0.9 V (high‐frequency resistance (HFR) corrected) under H_2_/O_2_ operation with flow rates of 1000 and 3000 sccm, respectively, for 15 min. Polarization curves were obtained by applying a series of potentiostatic steps from 0.4 V to the open‐circuit voltage (OCV) under H_2_/air conditions (1000/3000 sccm). Each potential was held for 4 min while HFR was simultaneously recorded at 5 kHz.

ECSA measurements were conducted via CV in the potential range of 0.08–0.98 V at a scan rate of 50 mV s^−1^, at 35°C and oversaturated conditions (∼500% RH). Hydrogen (1000 sccm) was supplied to the anode, while the cathode was purged with nitrogen prior to measurements to remove residual oxygen; during data acquisition, the cathode flow was set to zero. Each CV measurement consisted of seven cycles. The double‐layer charging current was determined by averaging the current in the 0.4–0.45 V region and subtracted from the measured current. The hydrogen hydrogen underpotential deposition (HUPD) charge was obtained by integrating the corrected current between 0.1 and 0.4 V after normalization by the scan rate. The Pt surface area was calculated assuming a charge density of 210 µC cm^−^^2^ for a monolayer of adsorbed hydrogen and subsequently normalized by the Pt loading, as determined by XRF, to yield the ECSA.

Sheet resistance (R_s_) was estimated from electrochemical impedance spectroscopy (EIS) measurements. EIS spectra were collected using a 10 mA perturbation at 0.3 V over a frequency range of 40 kHz to 0.5 Hz under H_2_/N_2_ conditions (H_2_ anode/N_2_ cathode) at 80°C, 100% relative humidity (RH), and 150 kPa absolute pressure. Gas flow rates were maintained at 1000 sccm for the anode and 3000 sccm for the cathode side. The effective sheet resistance was extracted from the 45° region of the high frequency Nyquist plot using ZView software [60, 61].

Additional EIS measurements were performed under H_2_/air conditions using a 10% current perturbation over a frequency range of 10 kHz to 0.1 Hz at 80°C, 100% RH, and 150 kPa. Data were collected at current densities of 0.2, 1, 1.5, and 2 A cm^−2^. Equivalent circuit models (ECMs) were obtained by fitting the impedance data, representing the electrochemical system with components corresponding to different contributions, including ohmic resistance (*R*_hfr_), charge‐transfer resistance (*R*_ct_), and mass transfer resistance (*R*_mt_), as shown in Figures S12 and S17. The ECM additionally included elements to account for inductive behavior observed at high current densities, including a constant phase element (CPE) and inductance (L), to better represent the electrochemical response of the fuel cell [62].

### 8.17 Fuel Cell Durability Evaluation

Durability was evaluated using a heavy‐duty fuel cell catalyst AST following the protocol reported by the M2FCT consortium. The MEAs were subjected to 90,000 square‐wave voltage cycles between 0.6 and 0.95 V, with a 0.5 s ramp and a 2.5 s dwell at each potential. The AST was conducted under H_2_/N_2_ operation (200/200 sccm) at atmospheric pressure, 100% RH, and 80°C. The durability after AST was evaluated via polarization curves, ECSA, and MA, following the same procedures applied for the fuel cell performance evaluation.

### 8.18 STEM‐EDS Analysis

For transmission electron microscopy analysis, MEAs were embedded in epoxy and sectioned by diamond‐knife ultramicrotomy to a thickness of approximately 70 nm. Aberration‐corrected scanning transmission electron microscopy (STEM) was performed using a JEOL JEM‐ARM200F NEOARM operated at an accelerating voltage of 200 kV. High‐angle annular dark‐field (HAADF) images were acquired at a resolution of 1024 × 1024 pixels, with a pixel size of 94 pm and a dwell time of 16 µs per pixel, using DigitalMicrograph software (Gatan, Inc.).

Energy‐dispersive x‐ray spectroscopy (EDS) elemental maps (512 × 512 pixels) were collected and processed using JEOL Analysis Station software with standardless Cliff–Lorimer quantification.

## Author Contributions

Y.S.K. conceived the idea and supervised the project. X.W., E.J.P., S.G.L., J.S.S., and Y.S.K. coordinated the collaboration and oversaw research activities. S.M.A., C.F., and E.J.P. synthesized and characterized the materials (NMR, GPC, water sorption, and impedance). H.P. and K.C. conducted MEA preparation and fuel cell testing, J.C., and S.Y.K. performed electrochemical half‐cell measurements, P.B. performed impedance measurement. L.A. conducted TEM experiments. H.G., D.K., and S.G.L. conducted DFT calculations. H.P., E.J.P., and Y.S.K. wrote the manuscript with input from all authors. All authors discussed the results and contributed to manuscript revision.

## Conflicts of Interest

SMA, EJP, and YSK filed a US patent application (19/197,330) on May 2, 2025 related to sulfonated poly(fluorene) materials. The remaining authors declare no competing interests.

## Code Availability

No custom code or mathematical algorithm was used for this study.

## Supporting information




**Supporting File**: adma74561‐sup‐0001‐SuppMat.pdf.

## Data Availability

The data that support the findings of this study are available from the corresponding author upon reasonable request.
