# Supplementary material for: Adsorption‐Engineered Hydrocarbon Ionomers for Durable Proton‐Exchange Membrane Fuel Cells
Source: Adv Mater. 2026 Aug 12;38(57):e74561. doi: 10.1002/adma.74561 (PMC13660832; doi:10.1002/adma.74561)
Supplement: Supplementary file 1 [file ADMA-38-e74561-s001.pdf]

Adsorption-Engineered Hydrocarbon Ionomers for Durable Proton-Exchange Membrane Fuel Cells

Heemin Park,[a,⊥] Kate Chen,[a,⊥] Su Min Ahn,[a,⊥] Hengquan Guo,[b,⊥] Cy Fujimoto,[c] Jong-Ho Choi,[a,d] Lynda Amichi,[e] Danah Kim,[b] Seung Geol Lee,[b,f,*] Xiaojing Wang,[a] Jacob S. Spendelow,[a] Sun Young Kang,[a] Panagiotis Bexis,[a] Eun Joo S. Park,[a,*] and Yu Seung Kim[a,*]

[a] MPA-11, Los Alamos National Laboratory, Los Alamos, NM 87545, USA

[b] Department of Materials Science and Engineering, Ulsan National Institute of Science and Technology (UNIST), Ulsan 44919, Republic of Korea

[c] Nanoscale Sciences Department, Sandia National Laboratories, Albuquerque, New Mexico 87123, United States

[d] Department of Electrical and Energy Engineering, Kyungil Univeristy, Gyeongsan-si, Gyeongbuk 38428, Republic of Korea

[e] Center for Nanophase Materials Science, Oak Ridge National Laboratory, Oak Ridge, TN 37830, USA

[f] Graduate School of Semiconductor Materials and Devices Engineering, Ulsan National Institute of Science and Technology (UNIST), Ulsan 44919, Republic of Korea

[⊥] Equal contributions

Seunggeol.lee@unist.ac.kr; epark@lanl.gov; yskim@lanl.gov

Table S1. Total energy of sulfate, fluorene, and *m*-terphenyl adsorbed on the Pt(111) surface.

**Total Energy/eV**		**Top**	**FCC**	**HCP**	**Bridge**
$HSO_3^{\bullet-}$	-	-473.05	-473.18	-473.30	Move to FCC
m-terphenyl	parallel	-664.52	-665.22	-663.97	Move to HCP
	perpendicular	-664.11	-662.02	Move to FCC	-664.40
o-terphenyl	parallel	-662.01	-661.66	Move to FCC	Move to HCP
	perpendicular	-660.52	-661.04	-661.66	Move to HCP
Dimethyl fluorene	parallel	-635.84	-636.58	-635.94	Move to FCC
	perpendicular	-635.85	Move to Top	Move to Top	Move to Top

Table S2. Ionomer properties of F3S100, F7S100, and Low Mw SDAPP.

**Ionomer**	**M_n (kg mol^{-1})[a]**	**PDI[b]**	**IEC (meq g^{-1})[c]**	**Water uptake (%)[d]**	**Proton conductivity (mS cm^{-1})[e]**	**Contact angle (degree)[f]**
F3S100	14	2.6	2.8	74	4.3	31
F3S100	26	2.0		63	4.3	31
F3S100	49	1.6		67	3.6	46
F7S100	6.8	1.3	2.3	48	3.5	84
Low Mw SDAPP	39	2.5	3.6	169	14.5	49

[a] Obtained from GPC analysis of the corresponding precursor polymers. [b] Representing molecular weight distribution, M_w/M_n. [c] Determined by acid-base titration. [d] Measured at 30 °C, 97% RH in powder form. [e] Measured in solution form; 0.1 M dissolved in DMSO. [f] Measured on ionomer-coated GDEs.

Table S3. Half-wave potentials (V) of various thin film electrodes in 0.1 M H_2SO_4.

	**Nafion**	**Pemion**	**F3S100**
Cell 1	0.804	0.783	0.775
Cell 2	0.779	0.793	0.783
Cell 3	0.792	0.785	0.782
Average	0.792 ± 0.013	0.787 ± 0.005	0.780 ± 0.004

Table S4. ECSA ($m^2\ g^{-1}$) of Nafion, Pemion, and F3S55-bonded Vulcan carbon-supported cathode as a function of potential cycling during break-in.

Number of cycles	Nafion	Pemion	F3S55
1st	77.8	46.5	~0
2nd	74.4	21.1	6.8
3rd	72.8	27.4	19.3
4th	71.6	30.7	27.0
5th	70.3	33.2	32.0
6th	69.1	34.7	35.6
7th	67.6	35.3	38.2
8th	66.9	35.9	40.2
9th	66.1	39.1	41.6
10th	65.0	37.3	42.8
11th		37.3	43.8
12th		37.5	44.5

Table S5. Mass activity (A mg^{-1}) of Nafion, Pemion, and F3S55-bonded Vulcan carbon-supported cathode as a function of potential cycling during break-in.

**Number of cycles**	**Nafion**	**Pemion**	**F3S55**
1st	0.15	0.02	0.007
2nd	0.19	0.03	0.029
3rd	0.22	0.06	0.029
4th	0.22	0.08	0.027
5th	0.22	0.10	0.026
6th	0.23	0.10	0.026
7th	0.23	0.11	0.026
8th	0.23	0.12	0.025
9th	0.23	0.13	0.026
10th		0.13	0.026
11th		0.13	0.026
12th		0.14	0.025

Table S6. ECSA ($m^2 g^{-1}$) of Nafion, Pemion, and F3S55-bonded HSC-supported cathode as a function of potential cycling during break-in.

**Number of cycles**	**Nafion**	**Pemion**	**F3S55**
1st	81.7	61.1	47.9
2nd	78.9	52.9	49.6
3rd	77.5	50.9	49.4
4th	76.4	49.8	49.0
5th	75.3	48.3	48.6
6th	74.3	47.9	48.2
7th	73.5	45.9	47.8
8th	72.7	47.1	47.6
9th	71.9	47.1	47.3
10th	71.4	47.3	47.1
11th	70.9	46.7	46.9
12th	70.4	46.4	46.6

Table S7. Mass activity (A mg^{-1}) of Nafion, Pemion, and F3S55-bonded HSC-supported cathode as a function of potential cycling during break-in.

**Number of cycles**	**Nafion**	**Pemion**	**F3S55**
1st	0.45	0.05	0.21
2nd	0.48	0.07	0.21
3rd	0.50	0.12	0.21
4th	0.50	0.20	0.21
5th	0.51	0.26	0.21
6th	0.52	0.31	0.21
7th	0.53	0.35	0.21
8th	0.53	0.39	0.20
9th	0.53	0.41	0.20
10th	0.53	0.45	0.19
11th	0.54	0.45	0.20
12th	0.54	0.42	0.18

Table S8. Mass activity (A mg_{pt}^{-1}) of Nafion, Pemion, and F3S55-bonded HSC-supported cathodes before and after AST.

**Cathode ionomer**	**BOL**	**90K**	**% loss**
Nafion	0.54	0.23	57.4
Pemion	0.42	0.10	76.2
F3S55	0.21	0.13	38.0
Low Mw SDAPP	0.46	0.08	82.6
F3S100 (14k)	0.064	0.047	26.5
F3S100 (26k)	0.042	0.039	7.1
F3S100 (49k)	0.048	0.041	14.6
F7S100	0.085	0.057	32.9

Table S9. ECSA ($m^2\ g^{-1}$) of Nafion, Pemion, and F3S55-bonded HSC-supported cathodes before and after AST.

**Cathode ionomer**	**BOL**	**90K**	**% loss**
Nafion	68.4	26.3	61.5
Pemion	55.3	19.6	64.6
F3S55	46.6	18.1	61.1
Low Mw SDAPP	51.1	19.5	61.8
F3S100 (14k)	62.1	42.3	31.9
F3S100 (26k)	63.8	42.2	33.9
F3S100 (49k)	67.1	49.3	26.5
F7S100	58.7	35.9	38.8

Table S10. Number of break-in cycles during conditioning for HSC-and Vulcan carbon supported cathodes with different ionomers.

	Sample	Number of cycles
HSC-supported cathodes	Nafion	14
	Pemion	14
	F3S55	12
	Low MW SDAPP	20
	F3S100 (14k)	13
	F3S100 (26k)	13
	F3S100 (49k)	13
	F7S100	27
Vulcan carbon-supported cathode	Nafion	9
	Pemion	16
	F3S55	12

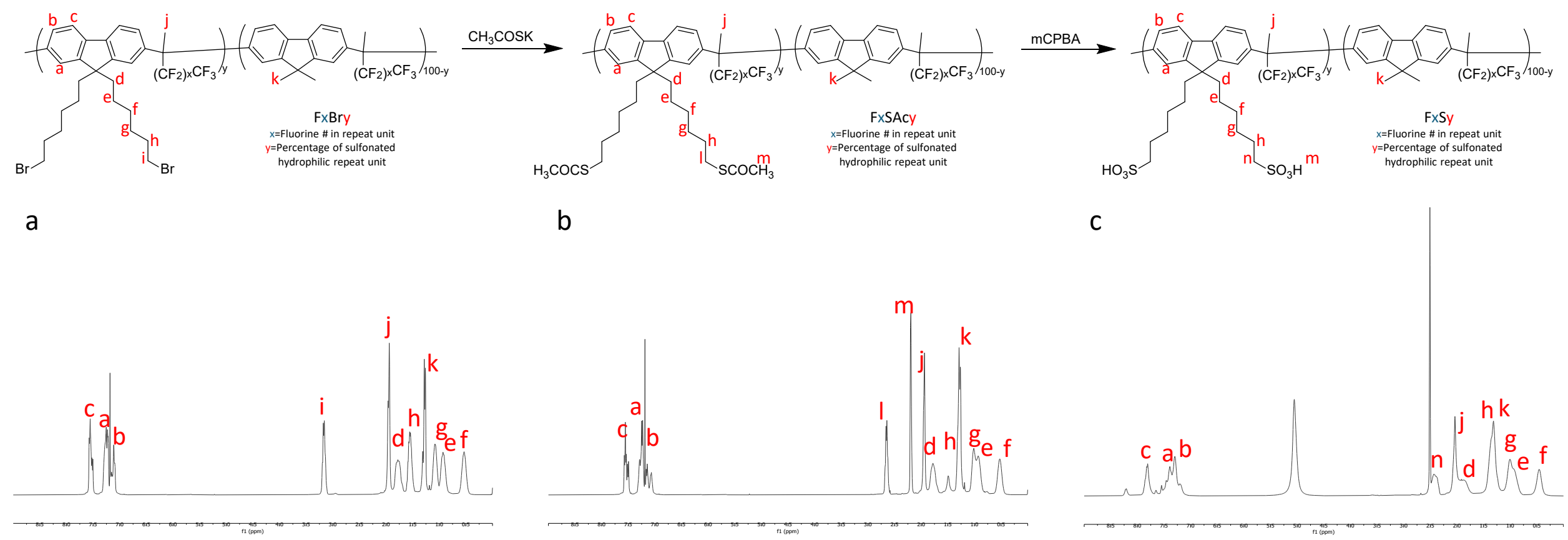

Figure S1| ^{1}H NMR spectra of (a) F3Br55 ($CDCl_3$), (b) F3SAc55 ($CDCl_3$) and (c) F3S55 (DMSO-*d*$_6$).

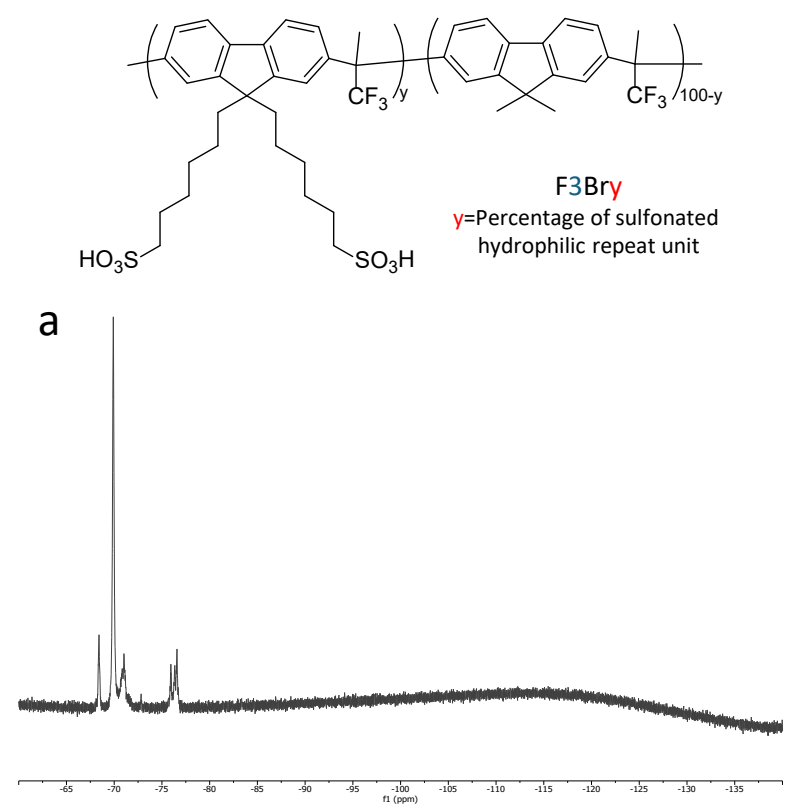

Figure S2| ^{19}F NMR spectrum of F3S55 in MeOH.

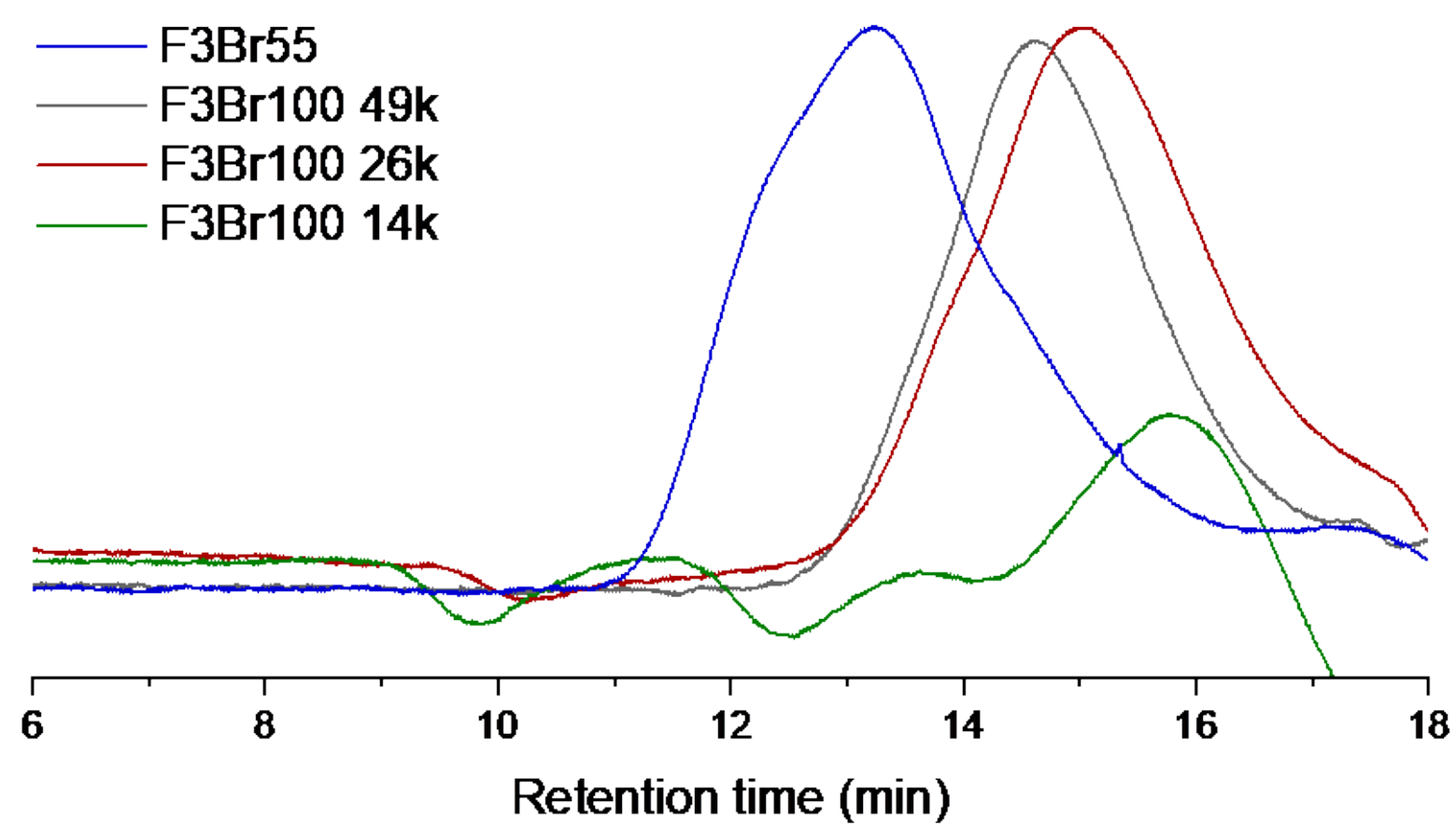

Ionomer	M_n (kg mol^{-1})	PDI
F3S55	102	2.8
F3S100	14	2.6
F3S100	26	2.0
F3S100	49	1.6
F7S100	6.8	1.3

Figure S3| GPC chromatograms and molecular weight of poly(fluorene) precursors.

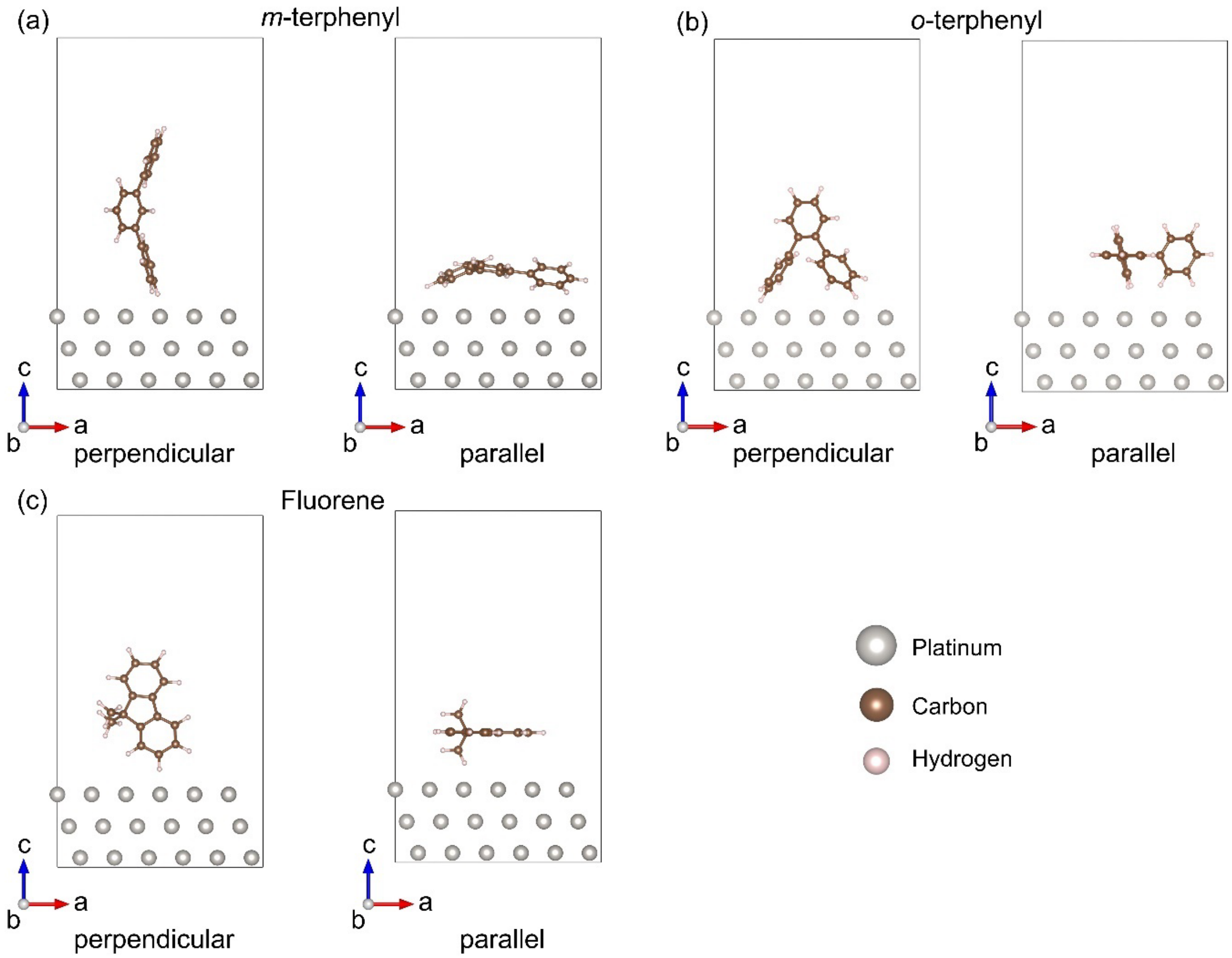

Figure S4| Schematic illustration of different adsorption orientations of aromatic molecules on the Pt(111) surface: (a) *m*-terphenyl, (b) *o*-terphenyl, and (c) dimethyl fluorene. For each molecule, perpendicular and parallel configurations are shown to the surface plane. Platinum, carbon, and hydrogen atoms are represented by gray, brown, and white spheres, respectively.

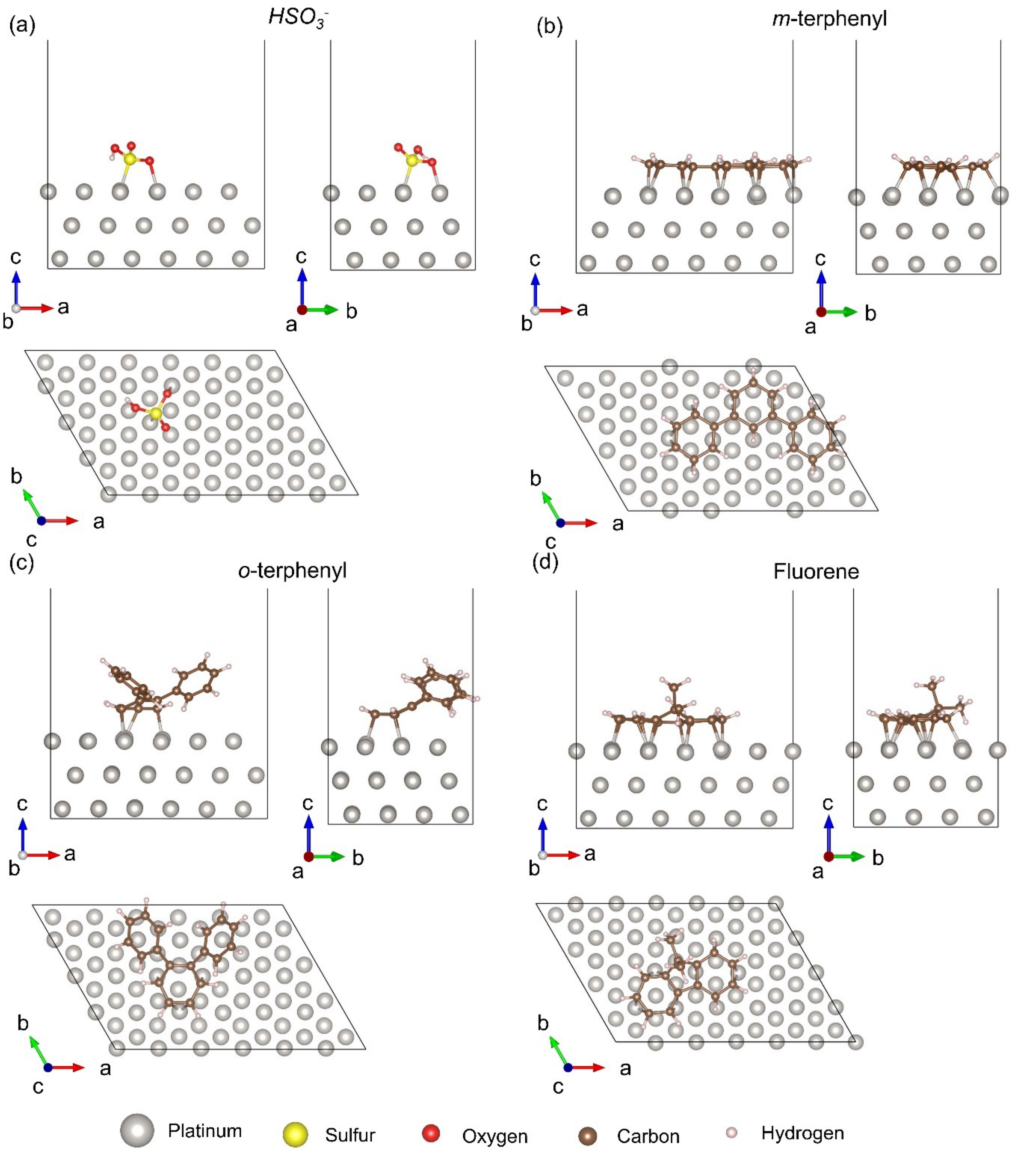

Figure S5| Optimized adsorption geometries of (a) HSO_3^-, (b) *m*-terphenyl, (c) *o*-terphenyl, and (d) dimethyl fluorene on the Pt(111) surface. For each adsorbate, side views along the a- and b-crystallographic directions, as well as the corresponding top view, are shown. Platinum atoms are depicted in gray, sulfur in yellow, oxygen in red, carbon in brown, and hydrogen in white.

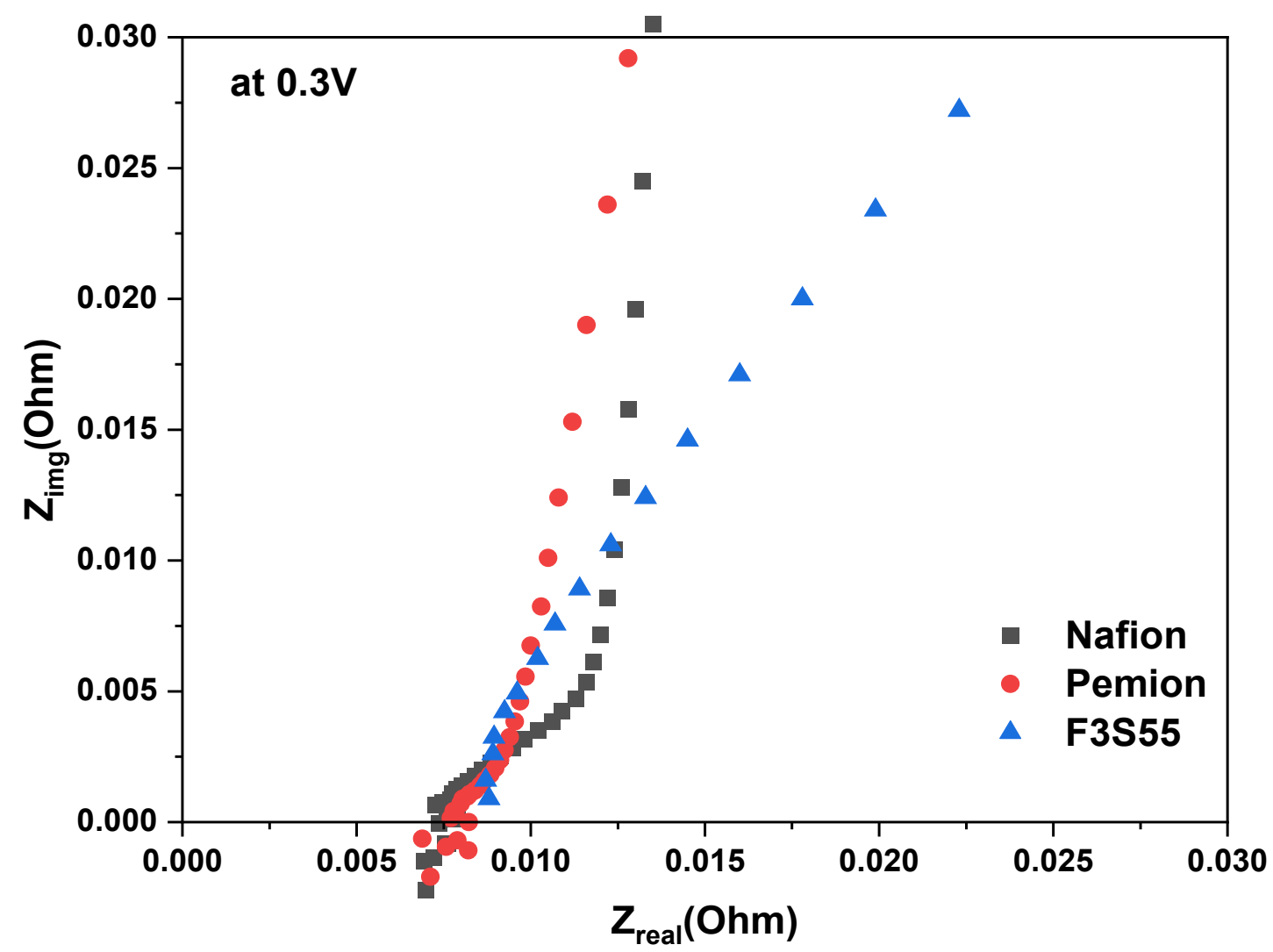

	R_s (ohm-cm^2)
Nafion	0.0166
Pemion	0.0074
F3S55	0.0703

Figure S6| Nyquist plots and sheet resistance (R_s) of various ionomer-bonded cathodes measured in H_2/N_2 configuration. Impedance measurements were performed using Vulcan carbon-supported Pt electrodes.

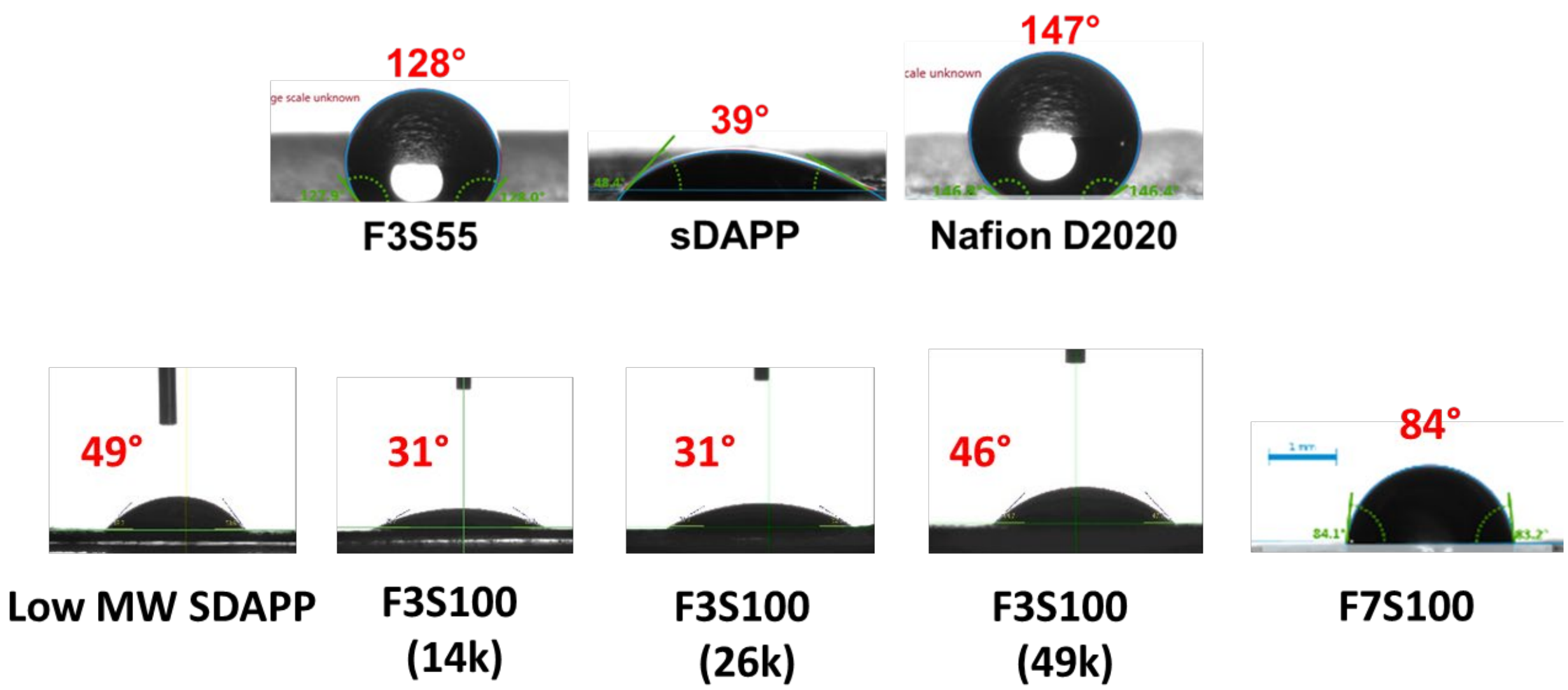

Figure S7| Water contact angle measurements on Pt/C gas diffusion electrodes (GDEs) coated with the ionomers. All contact angles were measured 60 seconds after the water was dropped.

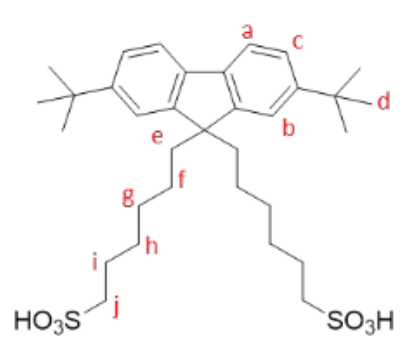

0.01 M, 60 mL in DI water

a : j = 2 : 3.48
Purity = (3.48/4)*100 = 87%

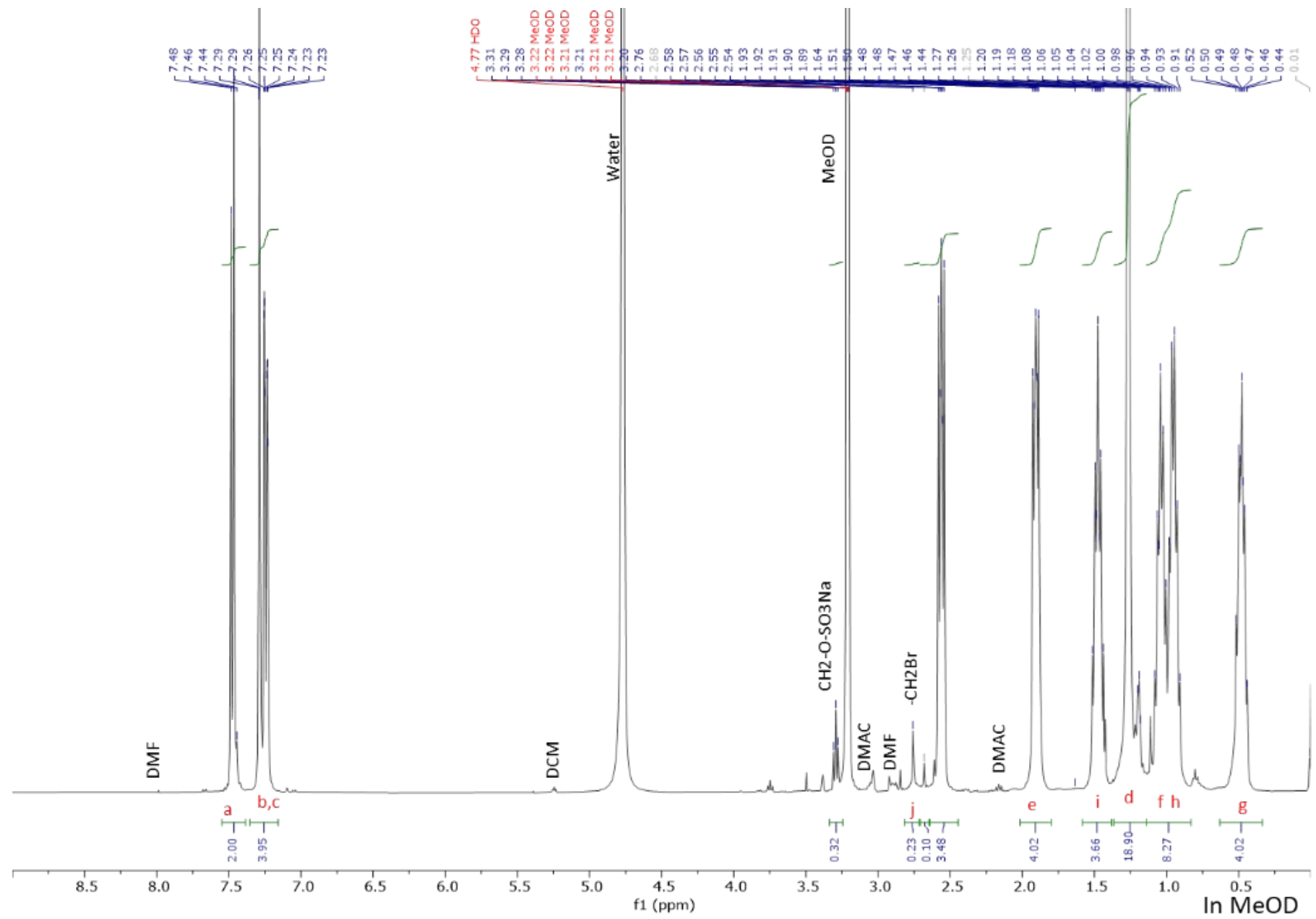

Figure S8| ^{1}H NMR spectrum of the 2,7-di-*tert*-9H-fluorene-9,9-dihexanesulfonic acid in D_2O.

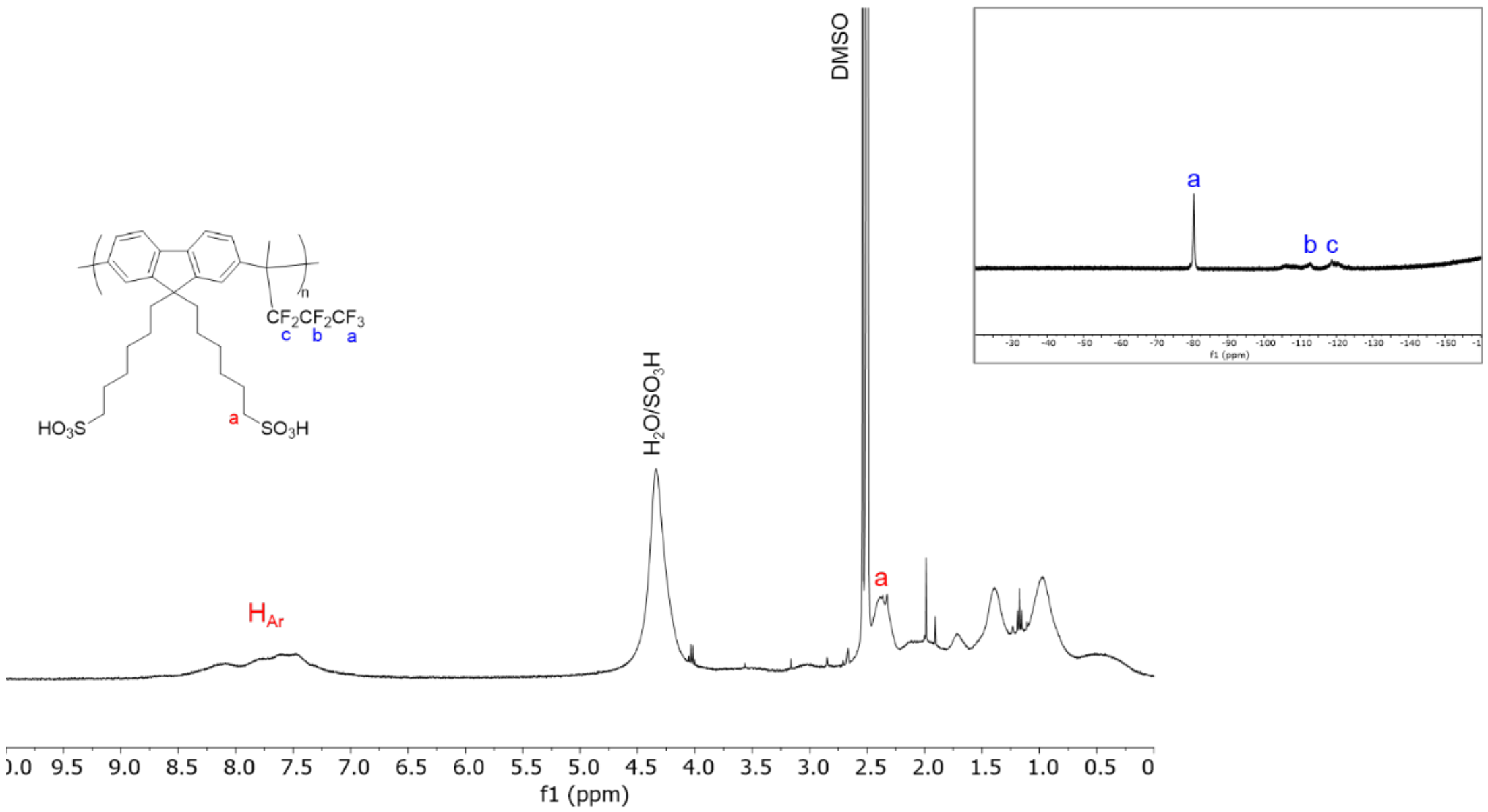

Figure S9| ^{1}H and ^{19}F NMR spectra of F7S100 in DMSO-d6.

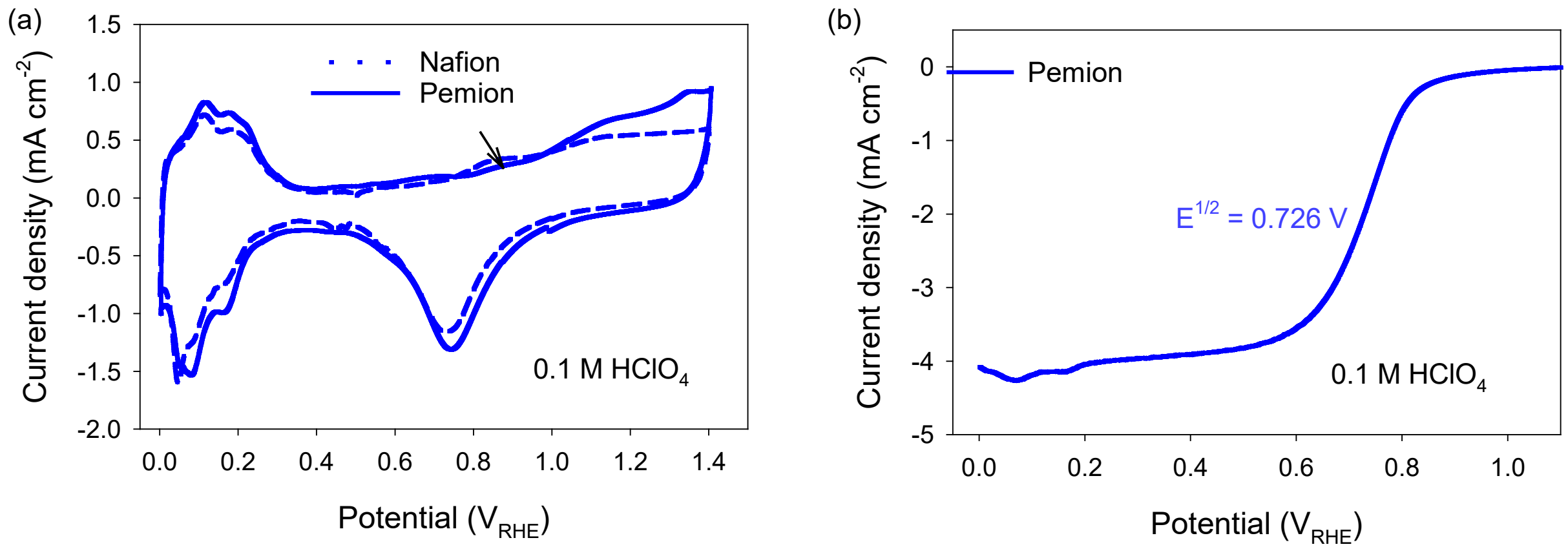

Figure S10| (a) CV comparison of Nafion and Pemion-bonded Pt/C electrodes in 0.1 M perchloric acid. The arrow indicates the onset of electrochemical oxidation current. (b) ORR voltammogram of the Pemion-bonded Pt/C electrode measured in 0.1 M perchloric acid.

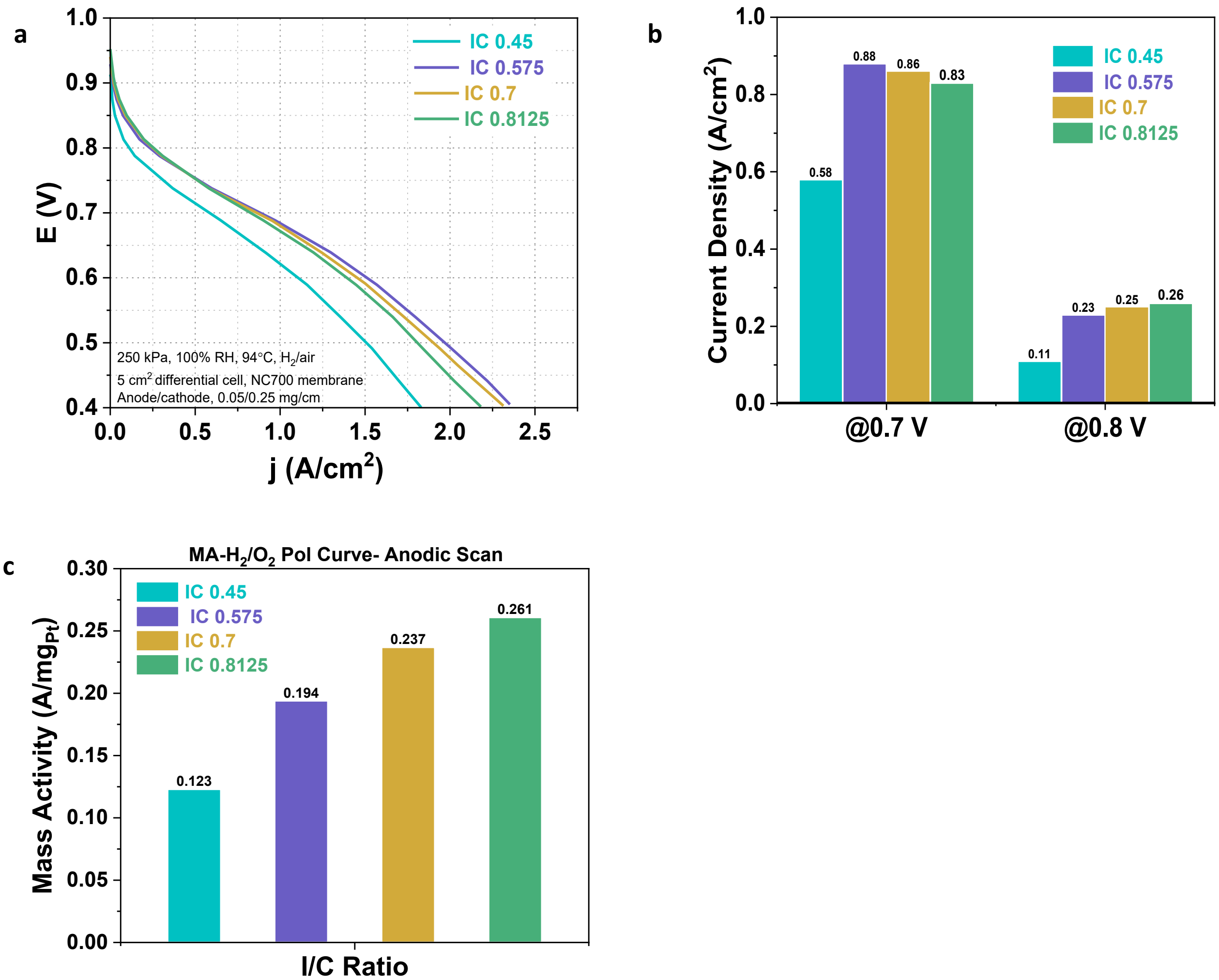

Figure S11| Optimization of Ionomer to catalyst (I/C) ratio for F3S55-bonded cathode (a) Polarization curves, (b) current density, and (c) mass activity as a function of I/C ratio. The cathode catalyst was TEC10E40E (37.6% Pt on HSC carbon), and the same catalyst was used at the anode. Catalyst loading is 0.25 mg cm^{-2} for the cathode and 0.05 mg cm^{-2} for the anode. The membrane was Chemours NC700. The ionomer was F3S55, and the I/C ratio varied from 0.45 to 0.8125, as indicated in the figure.

(a)

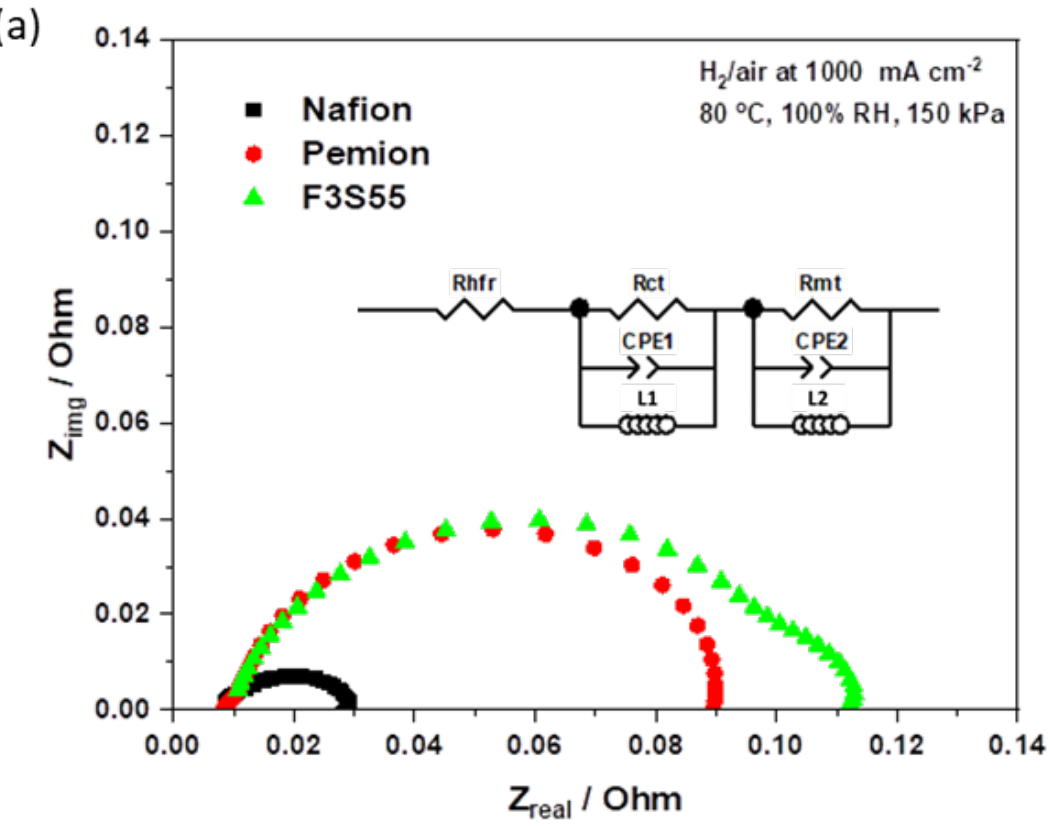

(b)

	R_{hfr} (Ω)	R_{ct} (Ω)	R_{mt} (Ω)
**Nafion**	0.008	0.002	0.020
**Pemion**	0.008	0.005	0.079
**F3S55**	0.010	0.009	0.094

Figure S12| EIS analysis of MEAs using Nafion-, Pemion-, and F3S55-ionomer-coated Vulcan cathodes. (a) Nyquist plots with the corresponding equivalent circuit model, (b) extracted high frequency (R_{hfr}), charge transfer (R_{ct}), and mass transfer resistance (R_{mt}) of the Vulcan cathodes.

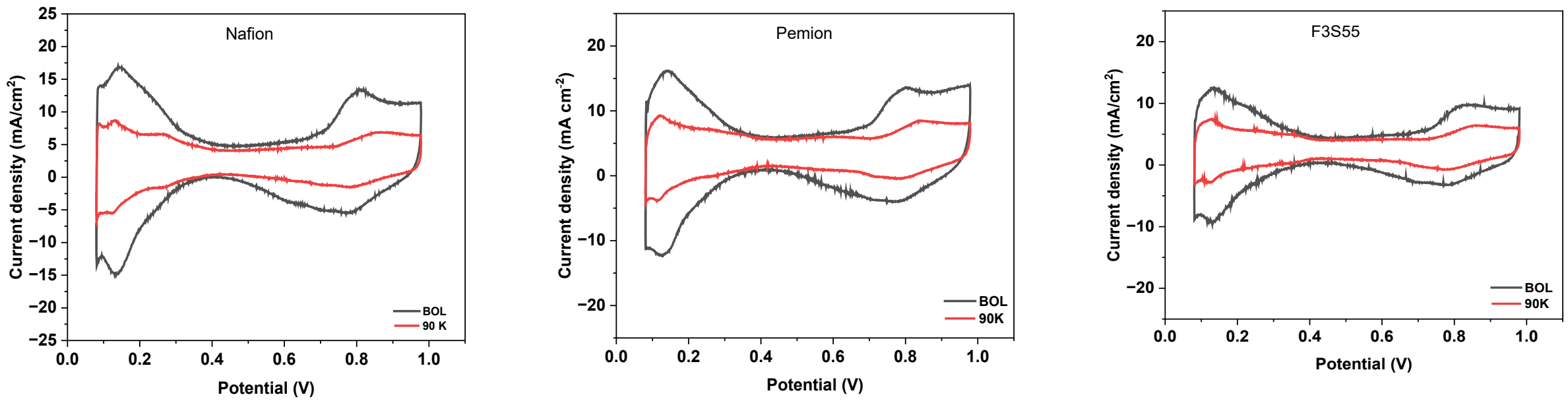

Figure S13| CV of Nafion, Pemion, and F3S55-bonded cathodes before and after AST.

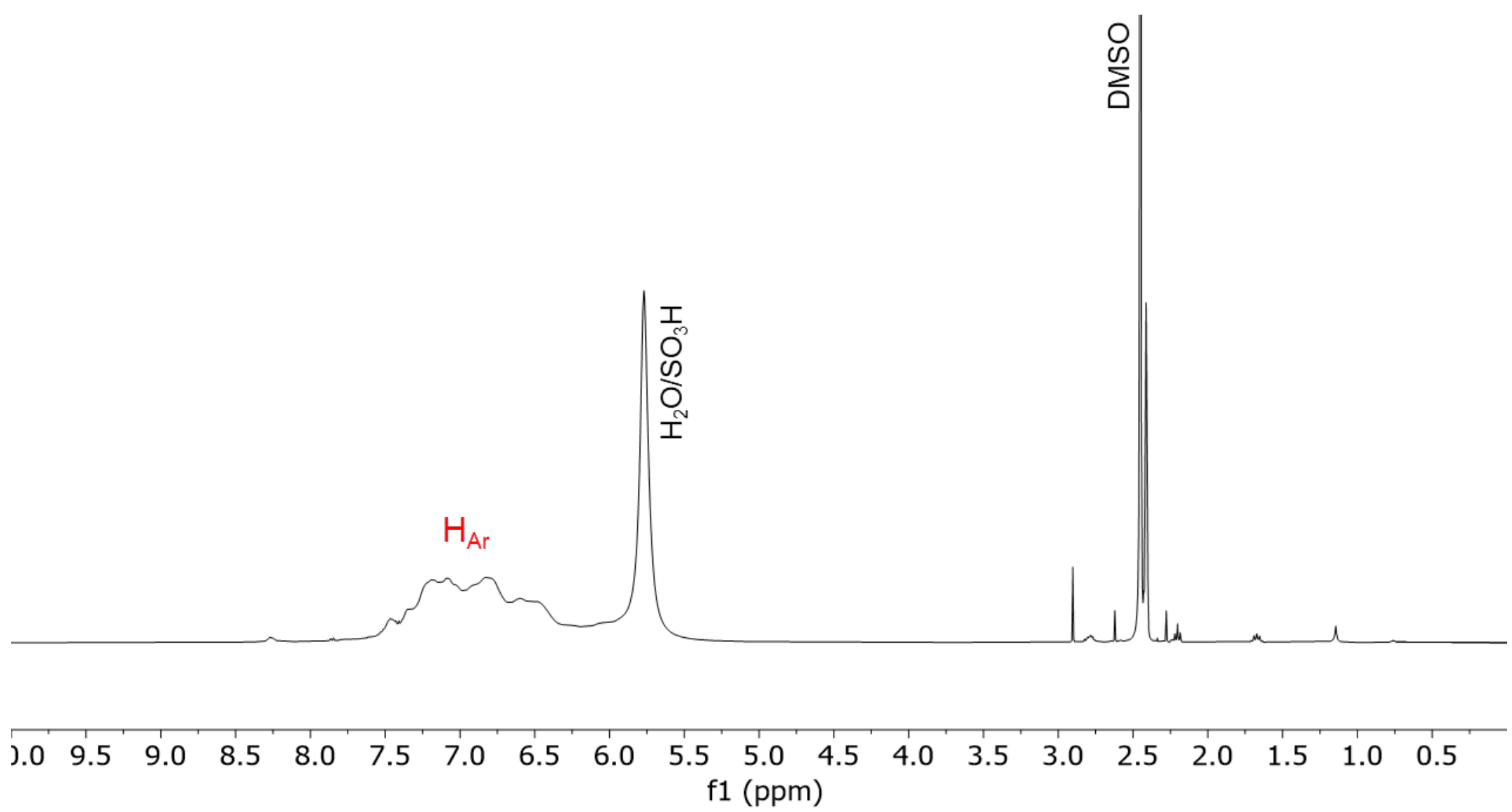

Figure S14| ^{1}H NMR spectrum of Low Mw SDAPP in DMSO-d_6.

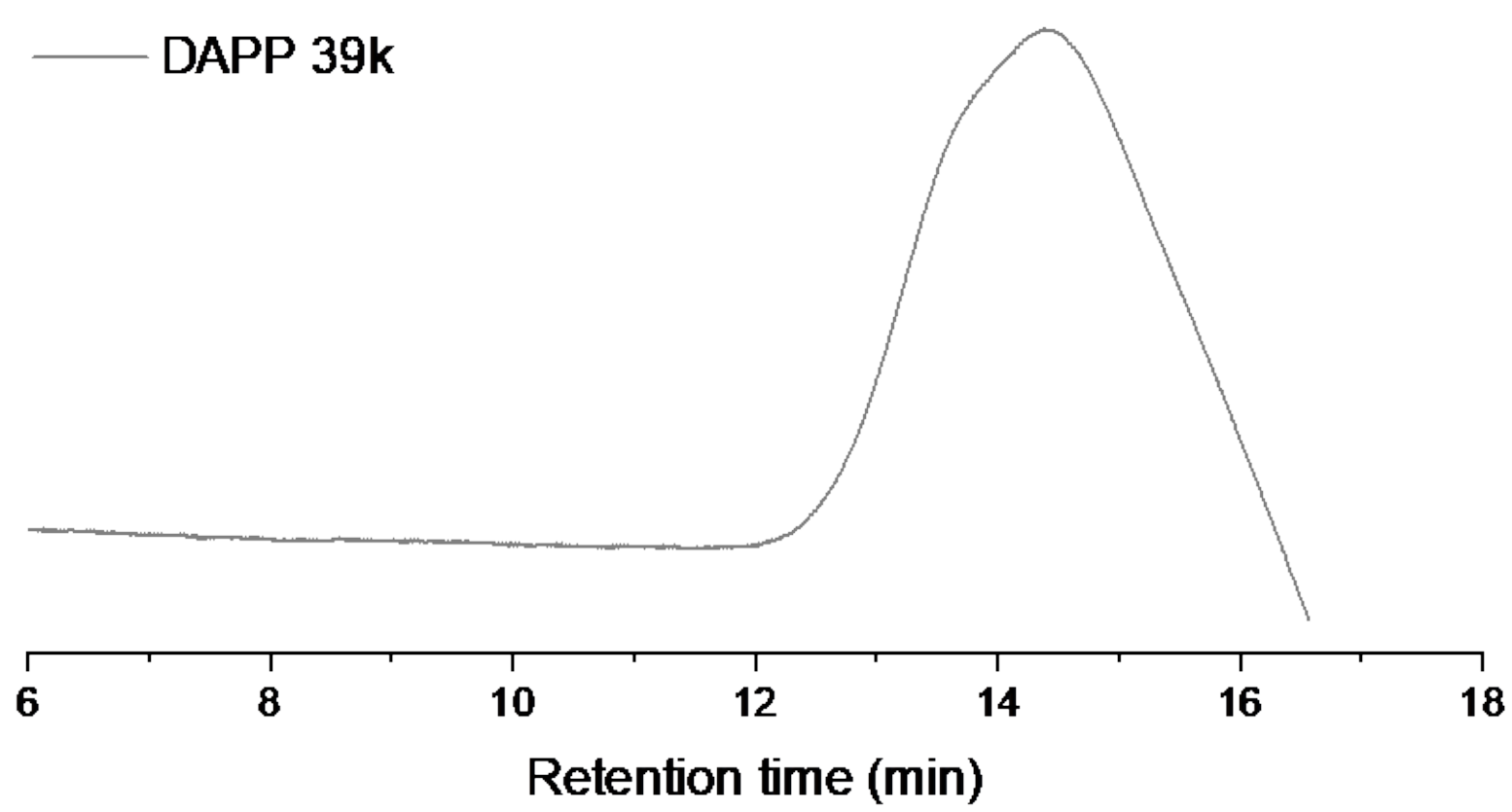

Figure S15| GPC chromatogram of Low MW DAPP (precursor of Low MW SDAPP).

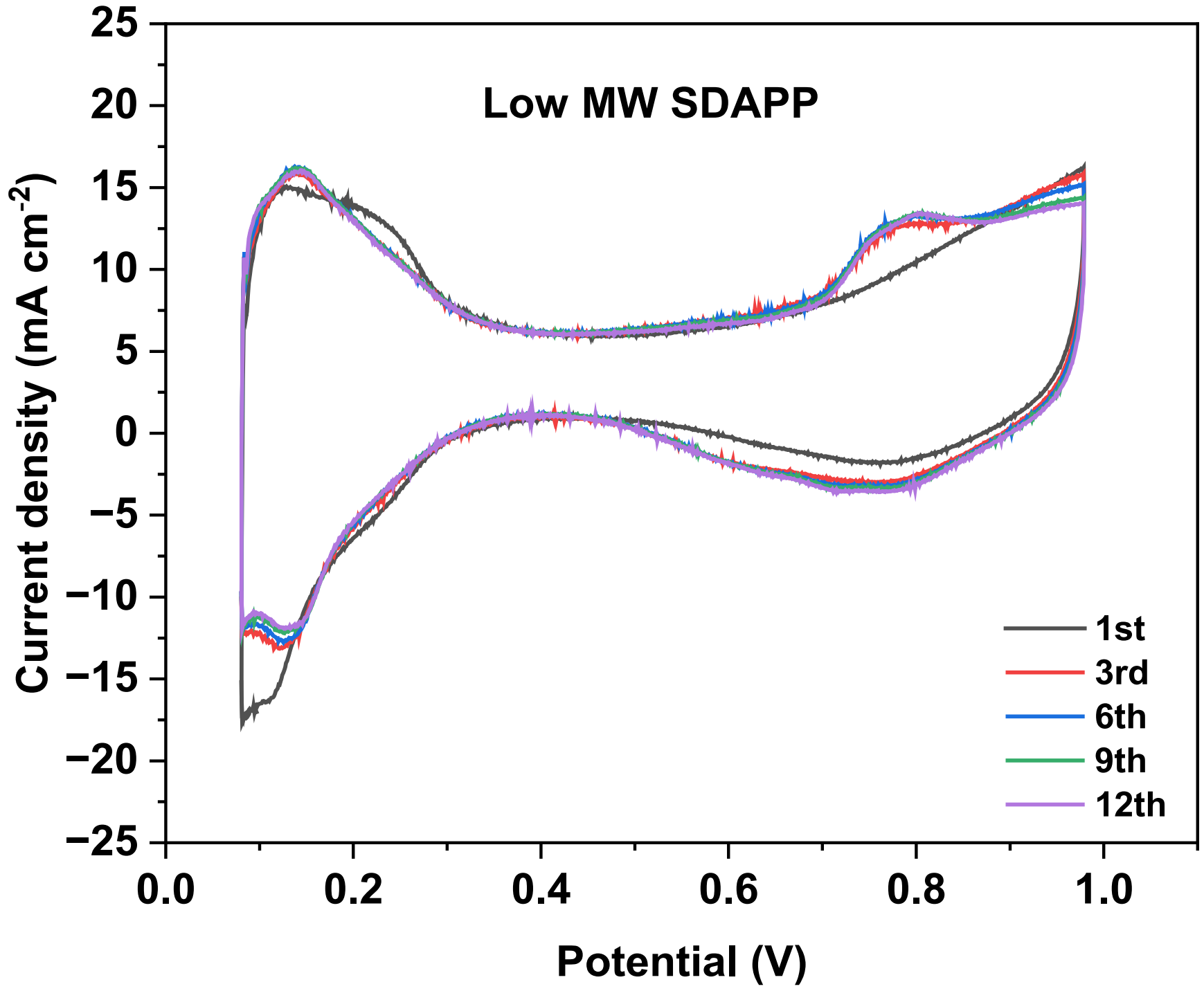

Figure S16| CV during break-in for Low MW SDAPP.

(a)

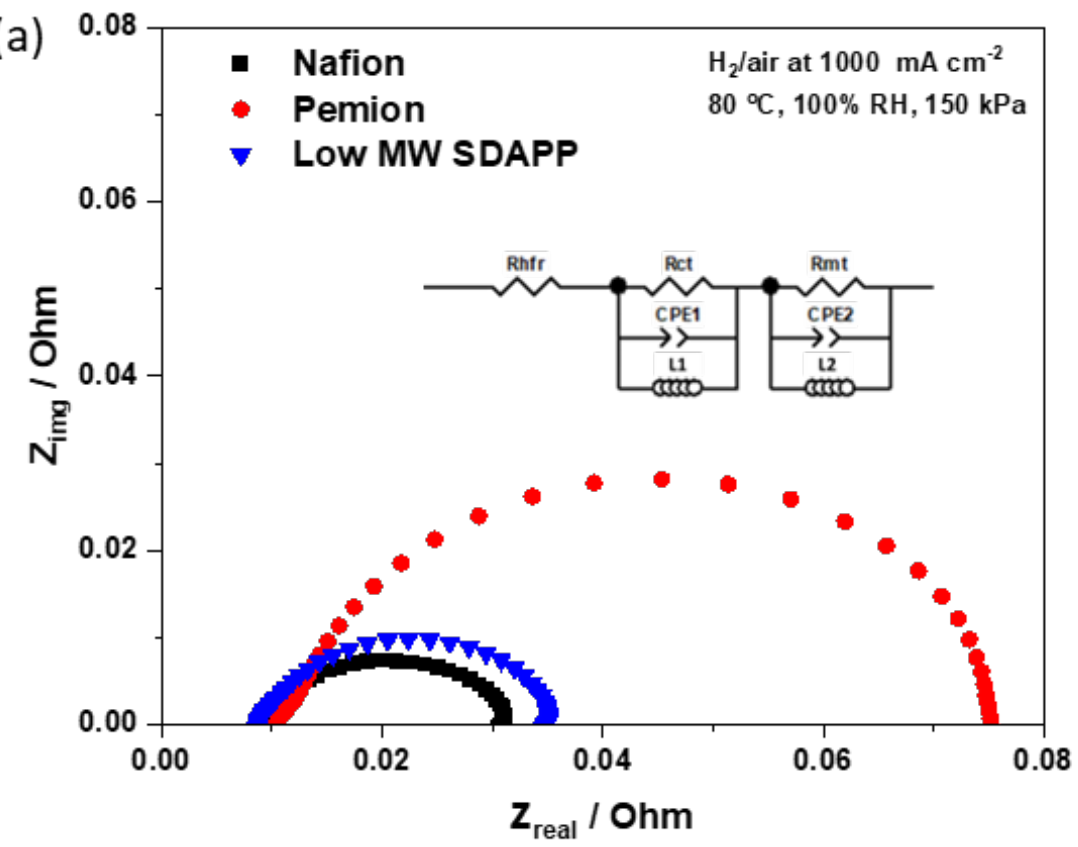

(b)

	R_{hfr} (Ω)	R_{ct} (Ω)	R_{mt} (Ω)
**Nafion**	0.009	0.002	0.022
**Pemion**	0.011	0.016	0.067
**low Mw sDAPP**	0.009	0.002	0.026

Figure S17| EIS analysis of MEAs using Nafion-, Pemion-, and Low MW SDAPP-ionomer-coated HSC cathodes. (a) Nyquist plots with the corresponding equivalent circuit model, (b) extracted high frequency (R_{hfr}), charge transfer (R_{ct}), and mass transfer resistance (R_{mt}) of the Vulcan cathodes.

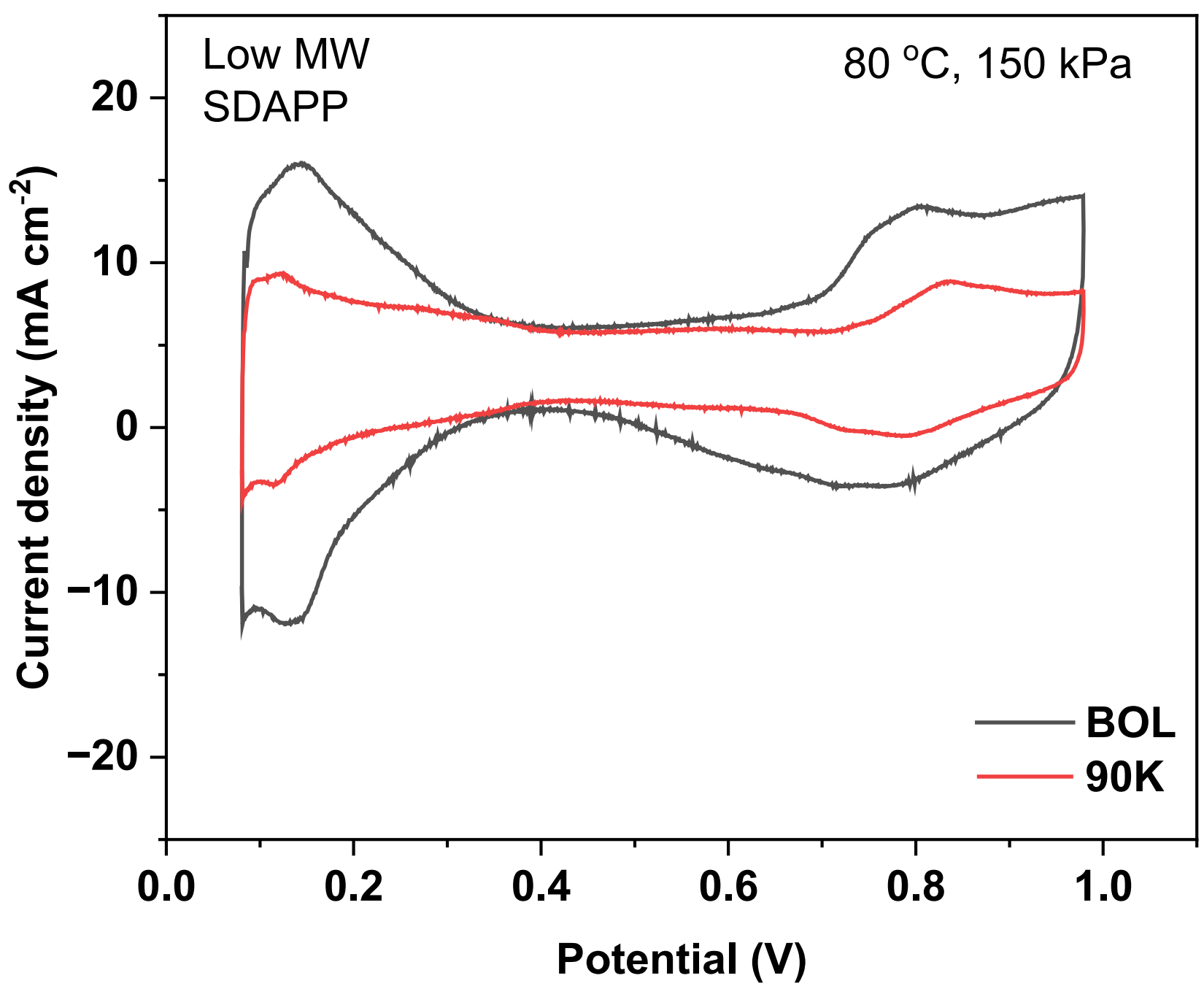

Figure S18| CV of Low MW SDAPP -bonded cathodes before and after AST.

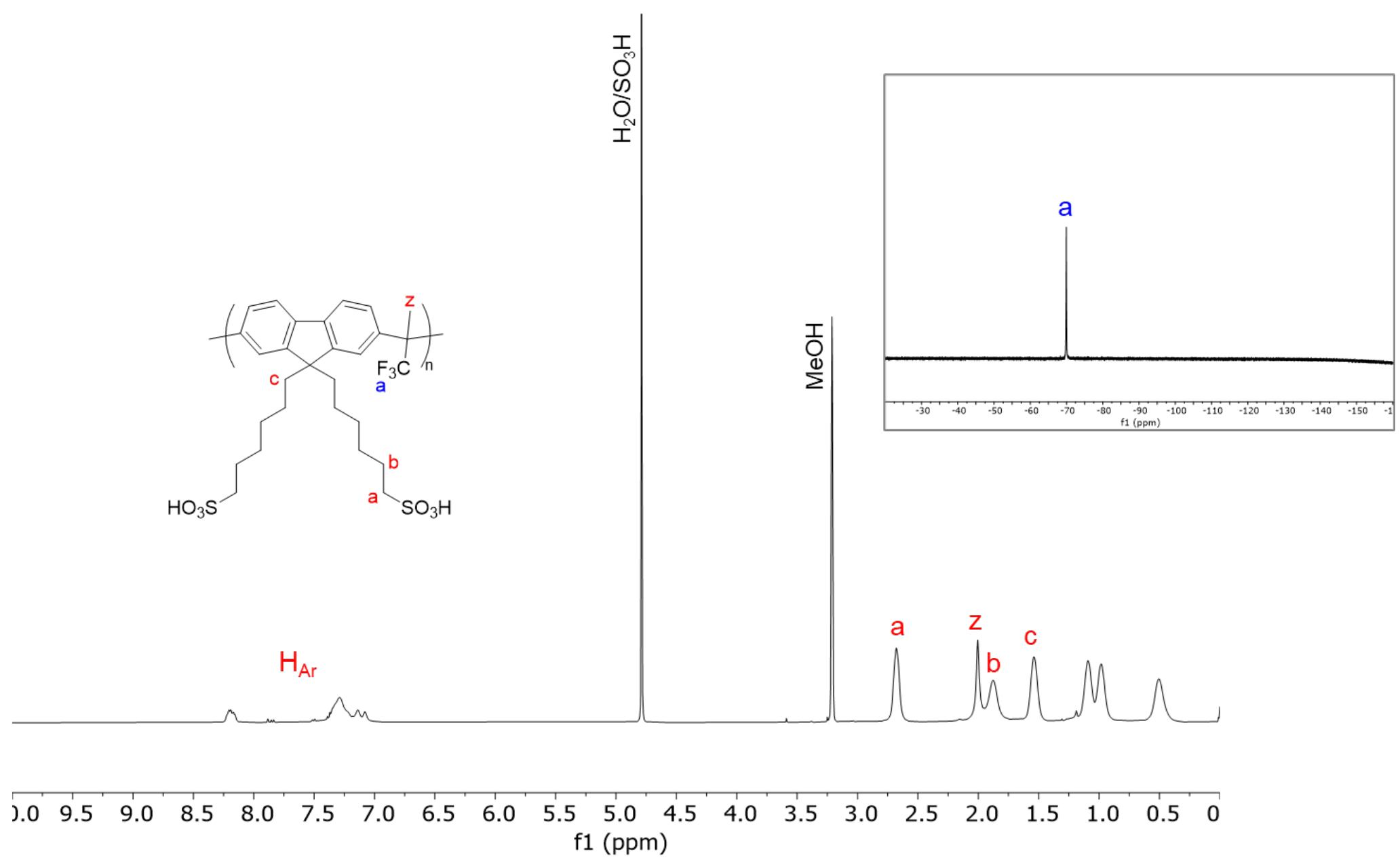

Figure S19| ^{1}H and ^{19}F NMR spectra of F3S100 in MeOH.

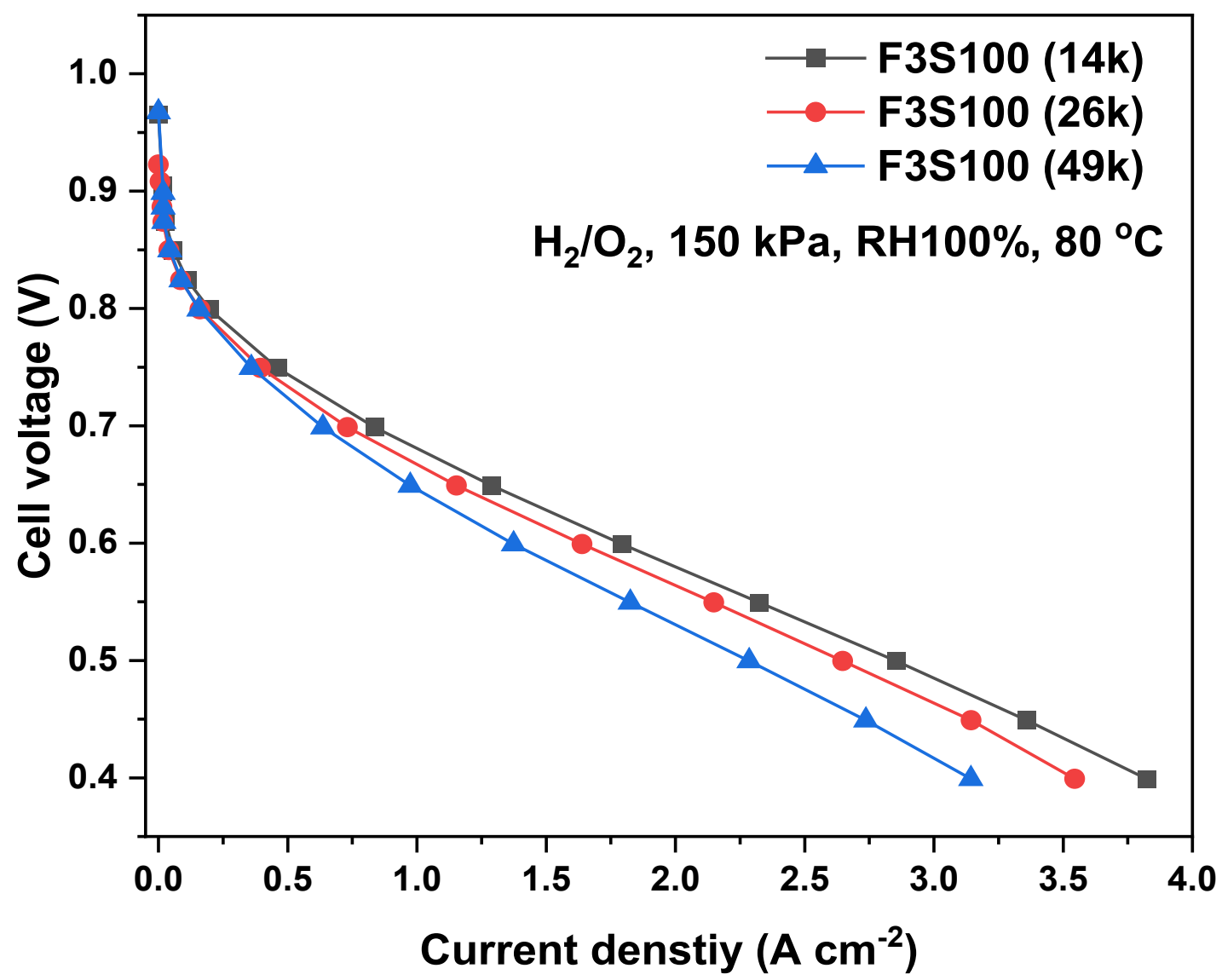

Figure S20| H_2/O_2 performance of F3S100-bonded cathodes.

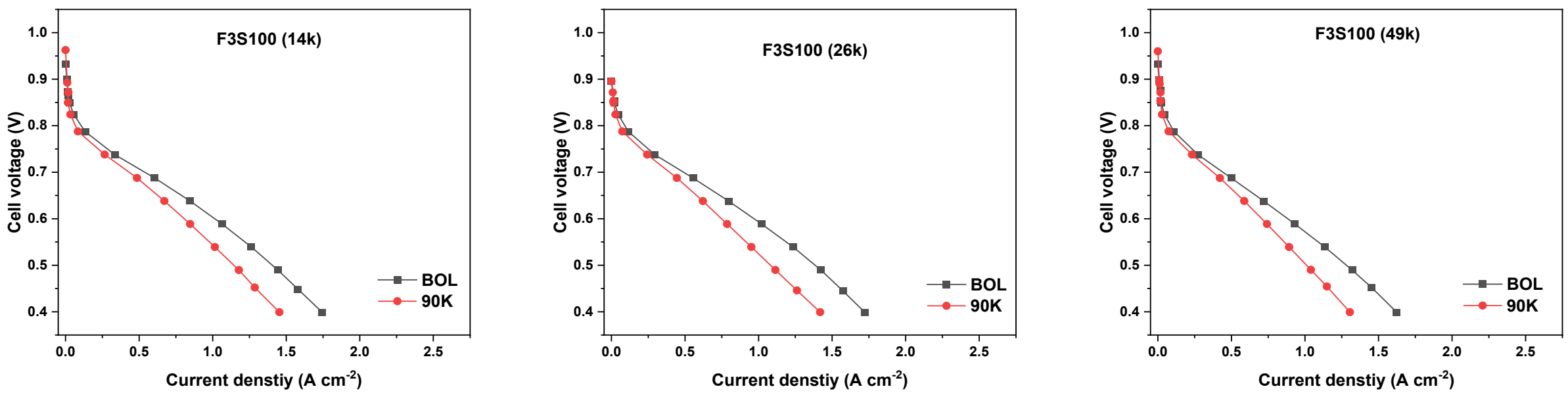

	Current density (A cm^{-2}) at 0.65 V		
M_n (kg mol^{-1})	14	26	49
BOL	0.85	0.80	0.72
90K	0.67	0.62	0.59
Loss %	21.2	22.5	18.1

Figure S21| Durability of F3S100-bonded cathodes. Polarization curves and current density at 0.65 V.

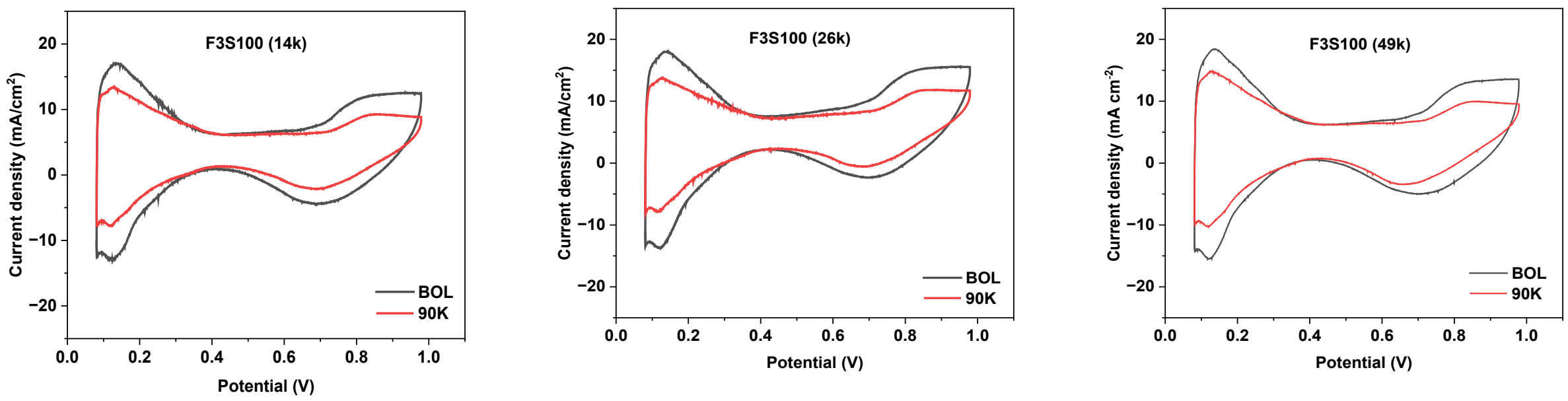

Figure S22| CVs of F3S100-bonded cathodes before and after AST.

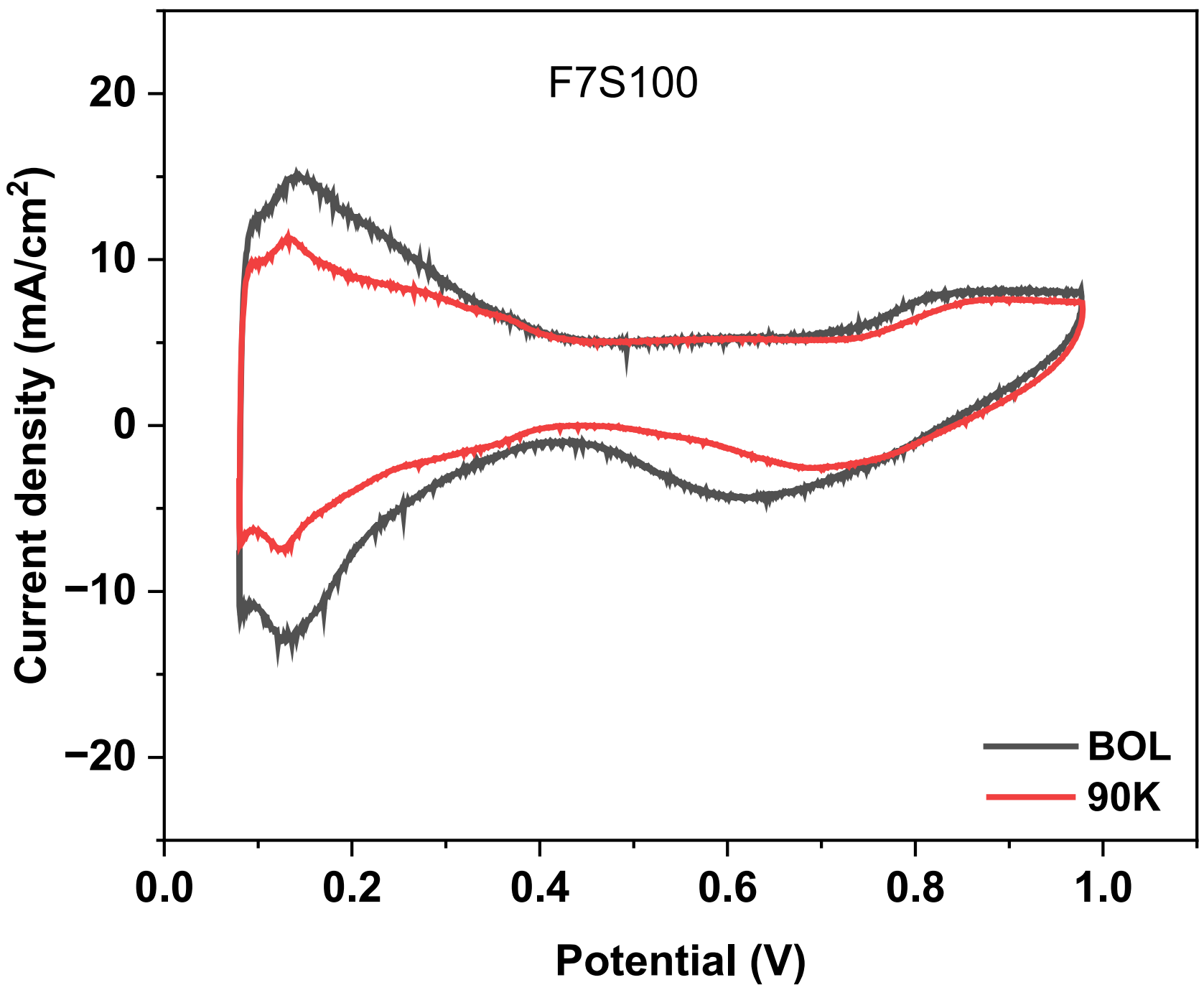

Figure S23| CVs of F7S100 -bonded cathodes before and after AST.

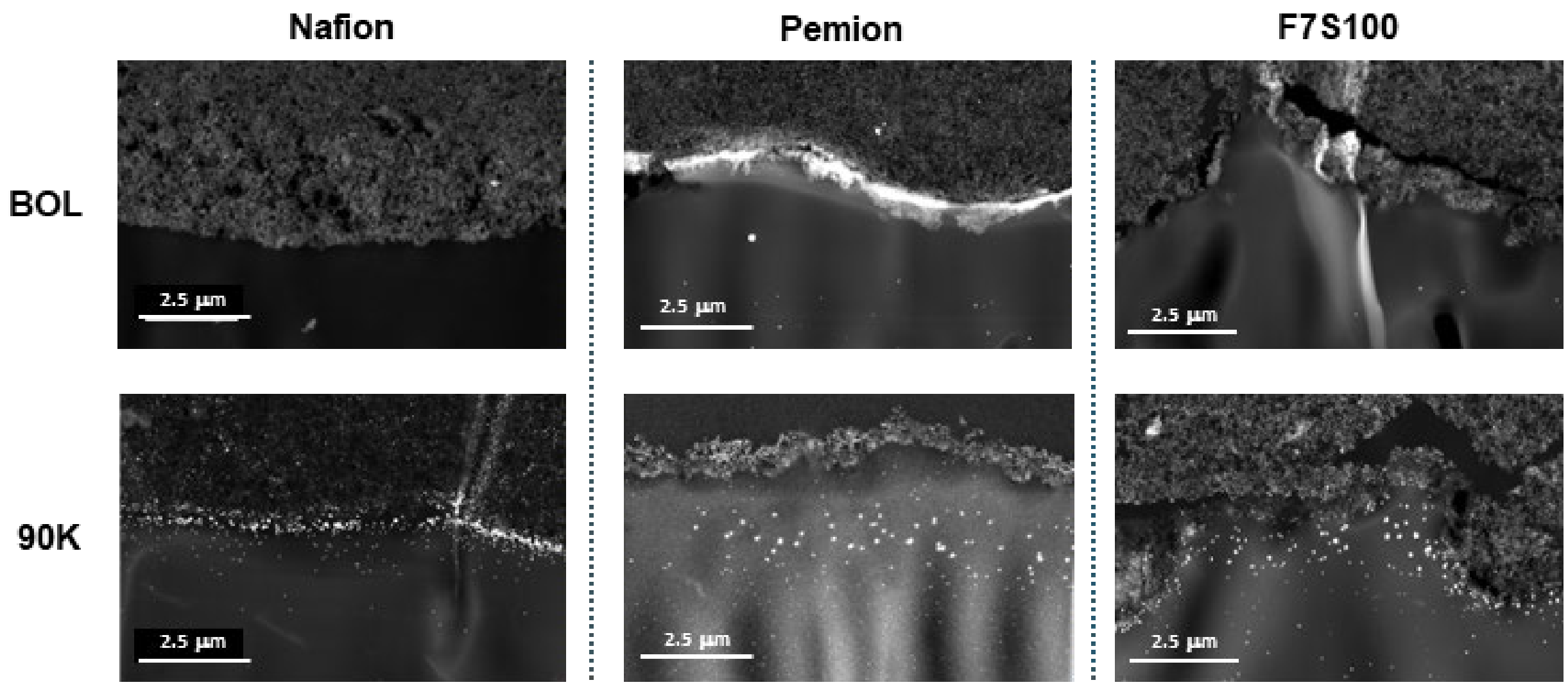

Figure S24| HAADF-STEM images of Nafion-, Pemion-, and F7S100-bonded cathodes before and after potential cycling AST.
